# Critical parameters for robust *Agrobacterium*‐mediated transient transformation and quantitative promoter assays in *Catharanthus roseus* seedlings

**DOI:** 10.1002/pld3.596

**Published:** 2024-06-05

**Authors:** Lauren F. Cole‐Osborn, Emma Meehan, Carolyn W. T. Lee‐Parsons

**Affiliations:** ^1^ Department of Chemical Engineering Northeastern University Boston Massachusetts USA; ^2^ Department of Chemistry and Chemical Biology Northeastern University Boston Massachusetts USA; ^3^ Department of Bioengineering Northeastern University Boston Massachusetts USA

**Keywords:** *Agrobacterium tumefaciens*, *Agrobacterium*‐mediated transformation, *Catharanthus roseus*, luciferase, transient expression, vacuum infiltration

## Abstract

*Agrobacterium*‐mediated transient expression methods are widely used to study gene function in both model and non‐model plants. Using a dual‐luciferase assay, we quantified the effect of *Agrobacterium*‐infiltration parameters on the transient transformation efficiency of *Catharanthus roseus* seedlings. We showed that transformation efficiency is highly sensitive to seedling developmental state and a pre‐ and post‐infiltration dark incubation and is less sensitive to the *Agrobacterium* growth stage. For example, 5 versus 6 days of germination in the dark increased seedling transformation efficiency by seven‐ to eight‐fold while a dark incubation pre‐ and post‐infiltration increased transformation efficiency by five‐ to 13‐fold. *Agrobacterium* in exponential compared with stationary phase increased transformation efficiency by two‐fold. Finally, we quantified the variation in our *Agrobacterium*‐infiltration method in replicate infiltrations and experiments. Within a given experiment, significant differences of up to 2.6‐fold in raw firefly luciferase (*FLUC*) and raw *Renilla* luciferase (*RLUC*) luminescence occurred in replicate infiltrations. These differences were significantly reduced when FLUC was normalized to RLUC values, highlighting the utility of including a reference reporter to minimize false positives. Including a second experimental replicate further reduced the potential for false positives. This optimization and quantitative validation of *Agrobacterium* infiltration in *C. roseus* seedlings will facilitate the study of this important medicinal plant and will expand the application of *Agrobacterium*‐mediated transformation methods in other plant species.

## 1 INTRODUCTION

*Agrobacterium*‐mediated transient transformation protocols have been developed for numerous non‐model and crop plants, serving as a quick and useful technique for probing gene function in vivo where methods for stable transformation are limited and labor‐intensive. In addition to model plants like *Arabidopsis thaliana* and *Nicotiana benthamiana*, recent examples of *Agrobacterium*‐mediated transient transformation protocols have been developed for spinach (Cao et al., 2017), rose (Lu et al., 2017), citrus (Acanda et al., 2021), avocado (Salazar‐González et al., 2023), quinoa (Xiao et al., 2022), apple (Lv et al., 2019), strawberry (Zeng et al., 2021; Zhao et al., 2019), and *Artemisia annua* (Li et al., 2021), among others. To ensure consistent and robust transformation, we characterized parameters critical for efficient *Agrobacterium*‐mediated transient transformation of *Catharanthus roseus* seedlings.

*C. roseus* has been studied for decades due to its unique ability to synthesize the complex terpenoid indole alkaloids (TIAs) vinblastine and vincristine. Researchers have characterized over 50 enzymes and transporters involved in TIA biosynthesis (recently reviewed in Kulagina et al., 2022) and multiple transcription factors that regulate TIA biosynthesis in response to defense hormones and environmental factors (e.g., Colinas et al., 2021). This foundational knowledge and the medicinal importance of its TIAs has transformed *C. roseus* into a burgeoning model organism for understanding plant specialized metabolism.

However, construction of transgenic *C. roseus* plants, a key tool for studying the genetics of model organisms, is still difficult and limited to only a few recent reports with low transformation and regeneration efficiencies (about 3% to 11%) and long timelines (about 4–6 months) (Bomzan et al., 2022; Choi et al., 2004; Kumar et al., 2018; Pan et al., 2012; Sharma et al., 2018; Verma et al., 2022; Verma & Mathur, 2011; Wang et al., 2012). Instead, researchers have relied on transgenic cell or tissue cultures (like cell, callus, and hairy root cultures) to study gene function (reviewed in Verma et al., 2017; Zárate & Verpoorte, 2007). These transgenic cultures illuminated the upstream TIA biosynthetic enzymes and their regulation, but the downstream TIA pathway leading to vinblastine and vincristine is only expressed in aerial tissue types and not in cell or hairy root cultures (Besseau et al., 2013; DeLuca et al., 1986; Dugé de Bernonville et al., 2020; Dutta et al., 2005; Góngora‐Castillo et al., 2012). Thus, transient methods of transforming these aerial tissue types such as virus‐induced gene silencing (VIGS) and *Agrobacterium* infiltration of aerial tissues have been developed in more recent years. VIGS in *C. roseus* leaf tissue has been pivotal in identifying TIA biosynthesis enzymes and regulatory factors (Carqueijeiro et al., 2015; Cruz et al., 2020; Liscombe & O'Connor, 2011; Yamamoto et al., 2021). Increasingly, *Agrobacterium* infiltration of *C. roseus* cotyledons, leaf tissue, and flower tissue has been used to overexpress transcription factors and monitor their effects on transcript levels or promoter activity, thus allowing rapid screening of transcription factor function (Carqueijeiro et al., 2021; Colinas et al., 2021; Colinas & Goossens, 2022; Di Fiore et al., 2004; Koudounas et al., 2022; Kumar et al., 2015; Kumar et al., 2018; Kumar et al., 2020; Mortimer et al., 2020; Pan et al., 2019; Schweizer et al., 2018; Van Moerkercke et al., 2016; Yang et al., 2023).

Our group has spent years developing a transient expression method in *C. roseus* seedlings known as the efficient *Agrobacterium*‐mediated seedling infiltration (EASI) method (Mortensen et al., 2022; Mortensen, Bernal‐Franco, et al., 2019). The EASI method uses vacuum infiltration to introduce *Agrobacterium tumefaciens* into *C. roseus* seedlings. The *Agrobacterium* contains plasmids expressing desired genes in their transfer DNA (T‐DNA) region. After infection, the T‐DNA will integrate randomly into the *C. roseus* genome and transiently express these desired genes, reaching a maximum expression after 3 days (Mortensen, Bernal‐Franco, et al., 2019).

Similar to flower or leaf‐infiltration, the EASI method is useful for studying transcription factor function in seedlings; one can overexpress a transcription factor and monitor transcript levels of TIA enzymes with qPCR. Additionally, we have successfully used the EASI method for promoter–reporter assays, which have previously only been performed in heterologous species like *N. benthamiana*. Thus, both promoter activity and transcript levels can be studied simultaneously in a native context using the EASI method. Studying promoter activity in a native context ensures that any necessary cofactors are present for promoter function and facilitates translation from transient to transgenic setting. For example, a previous report found significant differences between promoter activity in tobacco protoplasts compared with *C. roseus* cells. However, promoter activity in transiently transformed *C. roseus* cells was strongly correlated to stably transformed *C. roseus* cells (Van Der Fits & Memelink, 1997). Thus, studying promoter activity in *C. roseus* seedlings with our EASI method will provide results that are more likely to translate to fully transgenic *C. roseus* plants.

In our first iteration of the EASI method, we transformed *C. roseus* seedlings using an *Agrobacterium* co‐culture technique (Weaver et al., 2014). This method had limited transformation efficiency but exhibited the potential to transiently transform *C. roseus* seedlings. We significantly improved transformation efficiency by employing vacuum infiltration and optimizing multiple parameters, such as seedling age, *Agrobacterium* final optical density at 600 nm (OD_600_), number of days post‐infiltration prior to harvest, the use of a constitutively active *VirG* gene, and the use of silencing suppressors (Mortensen, Bernal‐Franco, et al., 2019). With this improved EASI method, we studied ORCA3 activation and ZCT1 repression of the *STR* promoter and *STR* expression; *STR* promoter activity and transcript levels increased similarly with *ORCA3* overexpression via EASI, highlighting the utility of EASI for studying both promoter activity and transcript levels (Mortensen et al., 2022). This EASI method was also used to study ZCT1 promoter activity (Mortensen, Weaver, et al., 2019).

Despite these successes, the transformation efficiency of the EASI method remained variable between experiments and sometimes luminescence fell below the limit of detection when weak promoters were studied. We increased the robustness of the EASI method by characterizing additional parameters that are critical for high transformation efficiency but not often tested. We additionally validated normalization methods and confirmed the quantitative nature of the dual‐luciferase assay in transiently transformed seedlings. This work will facilitate implementation of a useful tool for studying specialized metabolism in *C. roseus* and serve as a general resource for the development of *Agrobacterium*‐mediated transformation of other plant species.

## 2 METHODS

### 2.1 Cultivation and *Agrobacterium* infiltration of *C. roseus* seedlings

**FIGURE 1:**
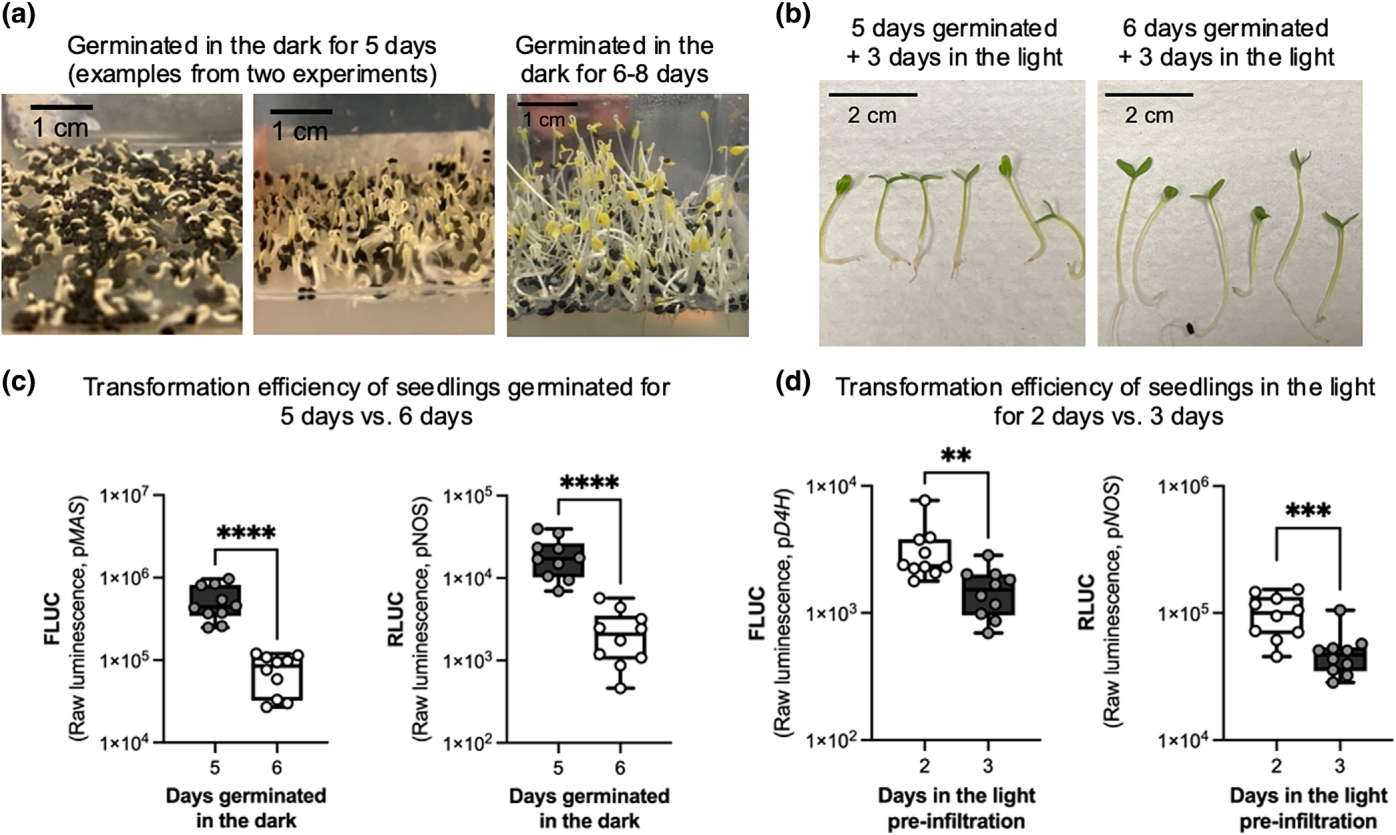
Seedling developmental state was critical for high transformation efficiency. (a) Seedlings germinated in the dark for 5 days were less than 1 cm in height with only the radicle and apical hook developed. In contrast, seedlings germinated in the dark for 6–8 days (our previous method) were 2–3 cm in height with cotyledons emerged. (b) After growth in light for 3 days, seedlings that had germinated in the dark for 5 days were shorter than seedlings germinated for 6 days. (c) Seedlings germinated in the dark for 5 days yielded higher luminescence, indicating higher transformation efficiency, than seedlings germinated for 6 days. (d) Seedlings germinated in the dark for 5 days and then grown in the light for 2 days had higher transformation efficiency than those grown in the light for 3 days. Transformation efficiency was measured by luminescence from firefly luciferase (FLUC) driven by the p*MAS* (c) or p*D4H* promoter (d) and luminescence from *Renilla* luciferase (RLUC) driven by the p*NOS* promoter. Each data point or biological replicate is a pool of two seedlings, *N* = 10. ****p < .0001, ***p < .001, **p < .01 according to an unpaired two‐tailed Student's *t* test on log‐transformed luminescence. Box plots represent the 25th and 75th percentile with a line marking the median. Whiskers extend to the minimum and maximum.

*C. roseus* var. Little Bright Eye seeds were surface sterilized as previously described (Mortensen et al., 2022). Seeds were carefully spread across the surface of the media without pushing them into the media; pushing seeds into the media can slow or inhibit germination (Figure S1). Seeds were germinated in the dark at 27°C for either 5 or 6 days to test the effect of seedling developmental stage (Figure 1a–c). After germination, seedlings were transferred to a 16‐h light/8‐h dark photoperiod under red and blue LED lights (about 100 μmol m^−2^ s^−1^) at room temperature (about 24°C). After 3 days in the light (Day 1: germinated seedlings were moved to light in the morning; Day 2: seedlings were in the light; Day 3: seedlings were in the light in the morning, moved to the dark in the afternoon), they were kept in the dark overnight (about 16–20 h) prior to infiltration.

To test the effect of light on seedling developmental stage, seedlings that were germinated in the dark for 5 days were grown in the light for 2 rather than 3 days (Figure 1d; Day 1: germinated seedlings were moved to light in the morning; Day 2: seedlings were in the light in the morning, moved to the dark in the afternoon for a pre‐infiltration dark incubation). To test the effect of the pre‐infiltration dark incubation, seedlings were germinated in the dark for 5 days, transferred to the light for 3 days, and then were either kept in light or transferred to the dark prior to *Agrobacterium* infiltration (Figure 3a).

Seedlings were vacuum infiltrated with *A. tumefaciens* GV3101 (pMP90) as previously described (Mortensen et al., 2022; Mortensen, Bernal‐Franco, et al., 2019). All experiments were infiltrated with two *Agrobacterium* strains in a 1:1 ratio at a final OD_600_ = .4 (OD_600_ = .2 per strain). One strain contained a reporter plasmid and the second strain contained a negative control effector plasmid expressing *beta‐glucuronidase* (*GUS*) (Figure S3). After infiltration, seedlings were placed in the dark for 2 days (post‐infiltration dark incubation) and then transferred to continuous light (red and blue LED lights, about 100 μmol m^−2^ s^−1^) at room temperature (about 24°C) for 24 h prior to harvest.

To test the effect of the post‐infiltration dark incubation, seedlings were either placed directly in a 16 h light/8 h dark photoperiod for 3 days (Figure 3b, no dark incubation) or in the dark overnight prior to starting a 16 h light/8 h dark photoperiod for 3 days (Figure 3b,c, overnight dark incubation). For comparison to the overnight dark incubation, seedlings were kept in the dark for 2 days and then placed in a 16 h light/8 h dark photoperiod for 1 day (Figure 3c, 2‐day dark incubation).

### 2.2 Golden Gate modular cloning

For each transient expression experiment, *C. roseus* seedlings were infiltrated with two *Agrobacterium* strains in a 1:1 ratio: one strain containing a reporter plasmid and the other containing an effector plasmid. All plasmids were constructed using Golden Gate Modular Cloning. Specific parts were obtained from the MoClo toolkit (Addgene Kit #1000000044) (Weber et al., 2011) or Moclo Plant Parts Kit (Addgene Kit #1000000047) (Engler et al., 2014) unless otherwise noted.

The reporter plasmid contained an intron‐containing firefly luciferase (*FLUC*) coding sequence (CDS) driven by either a strong constitutive promoter (p*MAS*) or a low‐expressing promoter (p*D4H*) and an intron‐containing *Renilla* luciferase (*RLUC*) CDS driven by a constitutive *A. tumefaciens NOS* (*AtuNOS)* promoter (Figure S3). The promoter and 5′UTR of *D4H* (approximately 1 kb upstream of the start codon) was amplified using Phusion High‐Fidelity DNA Polymerase (New England BioLabs) from *C. roseus* var. Little Bright Eye gDNA using primers listed in Table S1. The promoter and 5′UTR of *D4H* was cloned into the pICH41295 Level zero (L0) promoter + 5′UTR vector. The promoter + 5′UTR of *D4H* or the promoter + 5′UTR of *MAS* (pICH85281) was moved from L0 plasmids into the Level 1 vector (pICH47822) to drive the *FLUC* gene (containing plant‐specific introns; Mortensen, Bernal‐Franco, et al., 2019) with the *AtuOCS* terminator. This transcriptional unit was moved into the pSB90 backbone containing a second transcriptional unit that included the *RLUC* gene (containing plant‐specific introns; Mortensen, Bernal‐Franco, et al., 2019) under control of the *AtuNOS* promoter, *TMV* omega 5′UTR, and *AtuNOS* terminator. These two transcriptional units were arranged in a convergent orientation relative to each other (Figure S3A). Final L2 plasmids (p*D4H* reporter: pUN1309, Addgene Plasmid #203901; p*MAS* reporter: pSB138) were electroporated into *A. tumefaciens* GV3101 (pMP90).

The effector plasmid contained a *CaMV 2x35S* constitutively expressed *GUS* gene (pSB161, Addgene Plasmid #123197) (Figure S3B). For experiments varying EASI parameters, the *GUS* effector was not expected to activate the reporter gene but was included to mimic experimental conditions when the effector was a transcription factor candidate (Mortensen, Bernal‐Franco, et al., 2019).

### 2.3 Preparation of *Agrobacterium* cultures

**FIGURE 2:**
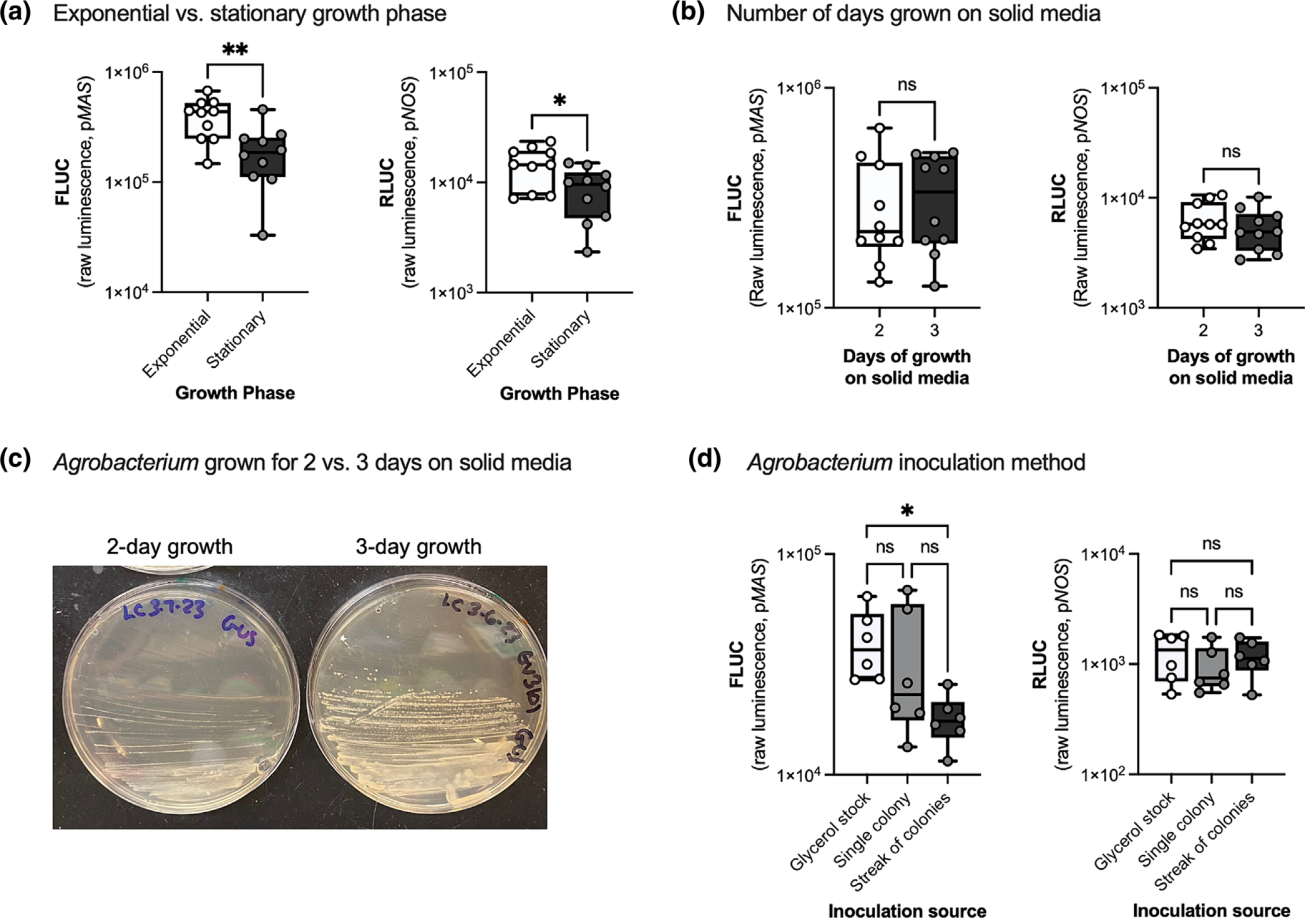
Variations in *Agrobacterium* growth conditions did not critically impact transformation efficiency. (a) *Agrobacteria* in exponential growth phase (OD_600_ = 1.0) increased transformation efficiency by about two‐fold compared with *Agrobacteria* in stationary phase (OD_600_ = 3.0). Transformation efficiency was measured by luminescence from firefly luciferase driven by the p*MAS* promoter (FLUC) and luminescence from *Renilla* luciferase driven by the p*NOS* promoter (RLUC). (b) The number of days that *Agrobacteria* was grown on solid media did not affect transformation efficiency. Glycerol stocks of *Agrobacteria* were streaked onto solid LB media containing selective antibiotics and were incubated at 25°C for 2 or 3 days before a streak of colonies was used to inoculate a liquid culture used for vacuum infiltration. (c) Visual comparison of streaked *Agrobacteria* on solid LB grown for 2 versus 3 days. Individual colonies were not visible after 2 days of growth. (d) Three different methods of cultivating *Agrobacterium* were comparable for transformation efficiency. Liquid *Agrobacterium* cultures were inoculated either directly from a glycerol stock or were first grown on solid media for 3 days before starting cultures either from a single colony or a streak of colonies. Each data point or biological replicate is a pool of two seedlings, *N* = 10. **p < .01, *p < .05 according to an unpaired two‐tailed Student's *t* test (a,b) or a one‐way ANOVA (d) on log‐transformed luminescence. Box plots represent the 25th and 75th percentile with a line marking the median. Whiskers extend to the minimum and maximum.

Prior to infiltration, *A. tumefaciens* GV3101 (pMP90) containing desired plasmids were streaked from frozen glycerol stocks onto solid LB media containing Gentamycin (10 mg/L Gent, selects for pMP90) and Kanamycin (50 mg/L Kan, selects for reporter or effector plasmid) and were grown at 25°C for 3 days. Only in Figure 2b was the *Agrobacterium* grown for 2 rather than the usual 3 days on solid media. For all experiments except those testing *Agrobacterium* growth conditions (Figure 2), a streak of colonies grown on solid media for 3 days was used to inoculate a 10 mL culture of LB with antibiotics (Gent and Kan) and grown overnight. The *Agrobacterium* was then induced and prepared for infiltration as previously described (Mortensen et al., 2022; Mortensen, Bernal‐Franco, et al., 2019).

To test the effect of *Agrobacterium* growth stage (Figure 2a), a single colony was grown in liquid LB media (3 mL of LB with Gent and Kan) overnight. After overnight growth, the OD_600_ of the culture was ~1.0–2.0. Either all 3 mL or 20 μL of this culture was used to inoculate 10 mL of culture and then grown overnight to achieve stationary or exponential phase (Figure 2a), respectively. After overnight growth, the stationary phase culture was OD_600_ = ~3.0, and the exponential phase culture was OD_600_ = ~1.0. The *Agrobacterium* was then induced and prepared for infiltration as previously described (Mortensen et al., 2022; Mortensen, Bernal‐Franco, et al., 2019).

To test the effect of *Agrobacterium* scale‐up method (Figure 2d), liquid media (3 mL of LB with Gent and Kan) was inoculated from either a single colony or directly from a glycerol stock. This culture was grown overnight and then used to inoculate a 10 mL culture of LB with Gent and Kan. The *Agrobacterium* was then induced and prepared for infiltration as previously described (Mortensen et al., 2022; Mortensen, Bernal‐Franco, et al., 2019).

### 2.4 Dual‐luciferase assay

Two seedlings were pooled for each biological replicate and protein was extracted and used in a dual‐luciferase assay, as described previously (Mortensen, Bernal‐Franco, et al., 2019). Luminescence was measured with a SpectraMax M3 plate reader in luminescence read mode (measuring all emission wavelengths) with an integration time of 500 ms. The limit of detection (LOD) was calculated as the blank + 3σ, determined by the average of at least eight empty wells, and samples were not analyzed if they were below the LOD.

## 3 RESULTS

### 3.1 Seedling growth parameters: transformation efficiency decreased as seedlings matured

We previously used our EASI method to study the regulation of the *STR* and *ZCT1* promoters (p*STR* and p*ZCT1*) in *C. roseus* seedlings (Mortensen, Bernal‐Franco, et al., 2019; Mortensen, Weaver, et al., 2019). However, this original EASI method could not be used to reliably study the *D4H* promoter (p*D4H*) with lower basal activity (Figure S2), resulting in a *FLUC* signal that was sometimes below the limit of detection (LOD: blank + 3σ). We thus aimed to increase the transformation efficiency of our EASI method to facilitate studying promoters with low activity. Developmental state of plant tissue is known to be an important factor influencing transformation efficiency. For example, we previously showed that 10‐day‐old seedlings were significantly more susceptible to EASI transformation than 14‐day‐old seedlings (Mortensen, Bernal‐Franco, et al., 2019). Here, we explored the influence of seedling developmental stage and showed that reducing time for germination in the dark and photomorphogenesis in the light further increased transformation efficiency.

First, we varied how long seedlings germinated in the dark. Germination time can vary considerably based on environmental conditions like temperature and humidity. Our original EASI method germinated seedlings for 7–8 days in the dark at 25°C which resulted in seedlings with unopened cotyledons and hypocotyls of around 1–2 cm in length (Mortensen et al., 2022; Mortensen, Bernal‐Franco, et al., 2019). Here, we condensed this timeline to 5 days of germination in the dark at 27°C. Under these conditions, only the radicle and the apical hook had emerged but not the cotyledons (Figure 1a). But after moving these seedlings to the light for 3 days, they showed expanded and green cotyledons. These seedlings that were germinated 5 days versus 6 days in the dark and then transferred to the light for 3 days only differed in having shorter hypocotyls (Figure 1b) and reduced germination rates.

We vacuum infiltrated these seedlings (germinated in the dark for 5 versus 6 days and then cultivated in the light for 3 days) with *Agrobacterium* containing a reporter plasmid encoding an intron‐containing *RLUC* driven by a constitutive *NOS* promoter and an intron‐containing *FLUC* driven by either a strong constitutive promoter (p*MAS*) or a low‐expressing promoter (p*D4H*) (Figure S3). When these constructs are used for promoter transactivation experiments, FLUC is measured as a reporter to study the activity of a promoter of interest. FLUC is divided by RLUC (FLUC/RLUC) to normalize for transformation efficiency. For these experiments studying transformation efficiency, we examined raw FLUC and RLUC luminescence. A factor that influences transformation efficiency is expected to influence both FLUC and RLUC raw luminescence. The p*MAS* reporter was used for most experiments to allow for a large quantitative range. The p*D4H* reporter was used when studying the effect of light conditions on the activity of a light sensitive promoter (Liu et al., 2019). An *Agrobacterium* strain containing a negative control effector plasmid, consisting of a constitutively expressed intron‐containing *GUS* gene, was co‐infiltrated in a 1:1 ratio with the reporter plasmid to mimic experimental conditions (Figure S3). FLUC and RLUC luminescence were measured 3 days after infiltration as a marker of transformation efficiency. Seedlings that had germinated for 5 days had a seven‐ to eight‐fold higher transformation efficiency compared with seedlings germinated for 6 days (Figure 1c), highlighting the critical importance of using young tissue for transformation.

Second, we reduced the number of days seedlings grew in the light prior to infiltration from 3 to 2 days. We again observed that younger seedlings transformed more efficiently—seedlings grown in the light for 2 days expressed significantly higher FLUC and RLUC than seedlings grown in the light for 3 days (Figure 1d). However, this effect was more moderate (approximately two‐fold) than the difference in transformation efficiency caused by changes to the germination timeline.

Overall, we showed that shorter developmental timelines using young seedlings for vacuum infiltration ensured the highest transformation efficiency. In addition to increasing transformation efficiency, these changes also reduced the length of time to complete an experiment.

### 3.2 *Agrobacterium* growth parameters: Virulence was robust despite variations in growth phase or cultivation method

We investigated the sensitivity of the EASI transformation efficiency with variations in *Agrobacterium* cultivation. To save time and labor, we investigated the effect of the growth phase (exponential vs. stationary) and the initiation and scale‐up method of *Agrobacterium* (starting from either a single colony, a streak of colonies, or directly from glycerol stock) on transformation efficiency.

In our original EASI method, we streaked *Agrobacterium* from glycerol stocks onto solid LB media with antibiotics for 3 days at 25°C and then started liquid cultures from a streak of colonies. This culture was grown overnight into stationary phase (OD_600_ = ~3.0) and then diluted to an optimized OD_600_ = .2–.4 for infiltration (Mortensen, Bernal‐Franco, et al., 2019). Growing the *Agrobacterium* into stationary phase resulted in three times the amount of *Agrobacterium* in the same volume, expediting experiments. However, when *Agrobacterium* reaches stationary phase, their viability decreases, potentially reducing transformation efficiency and uniformity. We thus tested the importance of using *Agrobacterium* in exponential versus stationary phase on EASI transformation. To conduct this study, we grew an *Agrobacterium* starter culture (3 mL) from a single colony overnight. We then started *Agrobacterium* cultures with varying volumes of the starter culture in order to catch the *Agrobacterium* in exponential phase (OD_600_ = ~1.0 (Nonaka et al., 2019; Semeniuk et al., 2014)) or stationary phase (OD_600_ = ~3.0). We confirmed that *Agrobacterium* in the exponential phase was more efficient at transforming *C. roseus* seedlings (Figure 2a). However, the difference was moderate (approximately two‐fold), so growing *Agrobacterium* into stationary phase reduced labor while still achieving high transformation.

In addition, we tested methods for initiating and scaling‐up the *Agrobacterium* culture. First, we tested *Agrobacterium* grown for only 2 versus 3 days on solid media before starting liquid cultures from a streak of colonies. With only 2 days on solid media, growth was observed but not individual colonies (Figure 2c). Using *Agrobacterium* grown for only 2 days on solid media led to a similar transformation efficiency as *Agrobacterium* grown for 3 days on solid media (Figure 2b), confirming flexibility in this timing.

We further reduced the *Agrobacterium* cultivation timeline by starting liquid cultures directly from glycerol stocks instead of from either a single colony or a streak of colonies grown on solid media. Similar transformation efficiency was observed between *Agrobacterium* started directly from glycerol stocks versus from a single colony or a streak of colonies grown on solid media (Figure 2d). Removing the intermediate step of growing *Agrobacterium* on solid media saves a considerable amount of time (3 days of growth) and labor (preparation of solid media).

Overall, the virulence of *Agrobacterium* was robust to alterations in growth conditions.

### 3.3 Infiltration parameters: Pre‐ and post‐infiltration incubations in the dark significantly increased transformation efficiency

Other environmental factors (such as light, temperature, humidity, and circadian rhythms) can influence *Agrobacterium* transformation efficiency (Azizi‐Dargahlou & Pouresmaeil, 2023; Cazzonelli & Velten, 2006; Zambre et al., 2003). The original EASI method included an overnight pre‐infiltration dark incubation of seedlings followed by a 2‐day post‐infiltration dark incubation. However, experimental evidence for these incubations was lacking in the literature.

**FIGURE 3:**
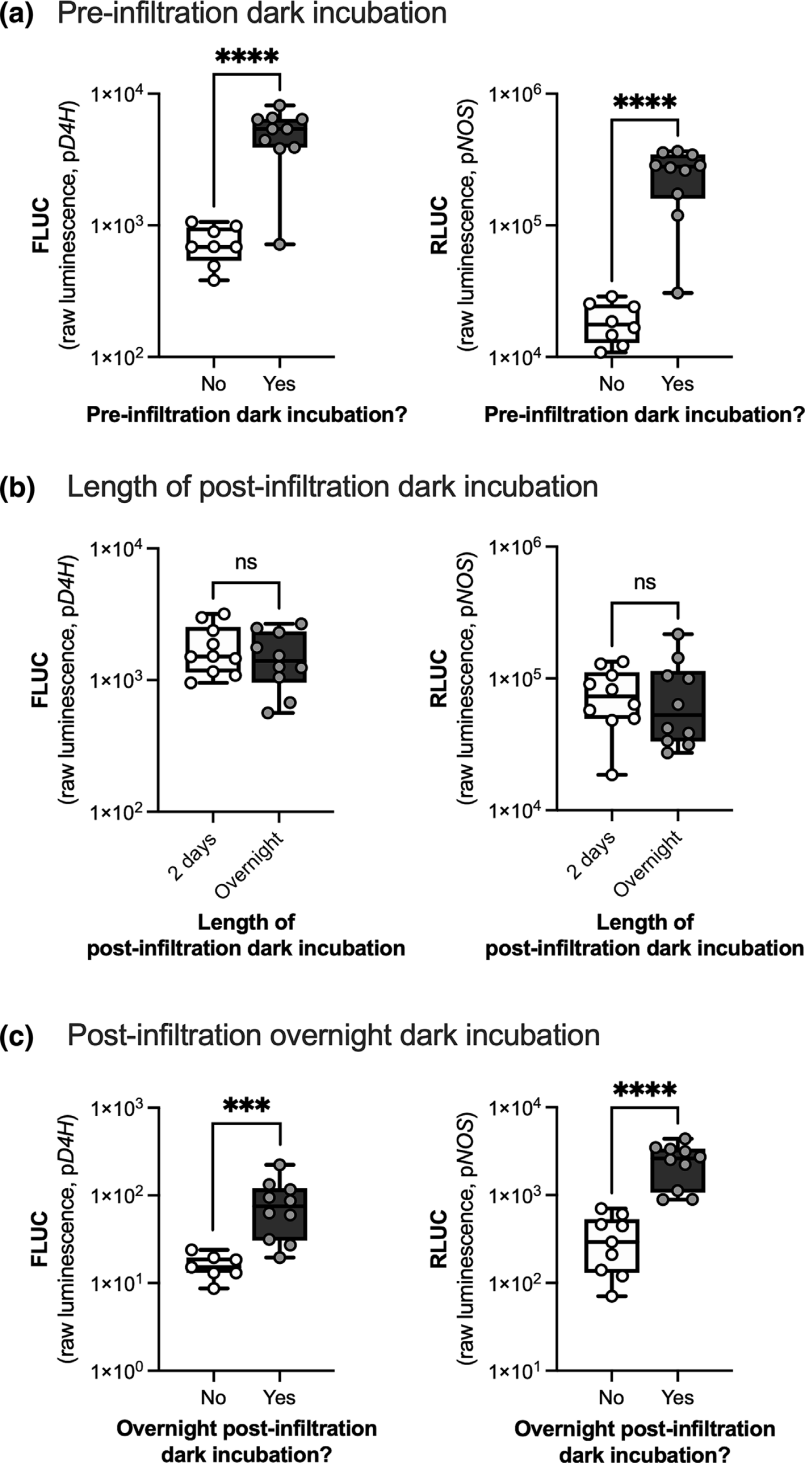
Dark incubations before and after infiltration were critical for high transformation efficiency. (a) Incubating the seedlings in the dark for about 18 h prior to infiltration (pre‐infiltration dark incubation) significantly increased transformation efficiency compared with no pre‐infiltration dark incubation. (b) Incubating the seedlings in the dark for 2 days rather than overnight post‐infiltration did not impact transformation efficiency. (c) Incubating the seedlings in the dark for about 18 h after infiltration (overnight post‐infiltration dark incubation) significantly increased transformation efficiency compared with no post‐infiltration dark incubation. Transformation efficiency was measured by luminescence from firefly luciferase driven by the light‐responsive p*D4H* promoter (FLUC) and luminescence from *Renilla* luciferase driven by the constitutive p*NOS* promoter (RLUC). Each data point or biological replicate is a pool of two seedlings, *N* = 10. ****p < .0001, ***p < .001 according to an unpaired two‐tailed Student's *t* test on log‐transformed luminescence. Box plots represent the 25th and 75th percentile with a line marking the median. Whiskers extend to the minimum and maximum.

We found that an overnight pre‐infiltration dark incubation is critical for high transformation efficiency, increasing luminescence seven‐ to 13‐fold compared with seedlings that did not undergo a pre‐infiltration dark incubation (Figure 3a). For post‐infiltration, we first tested whether the 2‐day post‐infiltration dark incubation could be shortened to an overnight dark incubation. There was no difference in transformation efficiency between the 2‐day and overnight dark incubation (Figure 3b). However, when the overnight post‐infiltration dark incubation was eliminated, a significant five‐ to seven‐fold decrease in transformation efficiency was observed, confirming that the post‐infiltration dark incubation is critical (Figure 3c).

For the above pre‐ and post‐infiltration dark incubation studies, we chose to use a light‐responsive promoter (p*D4H*) (Liu et al., 2019) to drive the expression of *FLUC* and a constitutive promoter (p*NOS*) to drive the expression of *RLUC* to understand how dark incubations affect both light‐responsive promoter activity and transformation efficiency. Because both FLUC and RLUC were affected by the dark incubations to a similar degree, these dark incubations seem to have a stronger impact on overall transformation efficiency rather than light‐responsive promoter activity.

### 3.4 Quantitative validation: Normalization to RLUC and a control condition effectively reduced variation and false positives

Transgene levels expressed via *Agrobacterium*‐mediated transformation are notoriously variable (Butaye et al., 2005; Peach & Velten, 1991; Sohn et al., 2011; Zeng et al., 2021). This could introduce confounding variability between conditions and preclude accurate conclusions from being drawn when evaluating the activity of an effector on a promoter of interest. To control for the variability of transformation efficiency, we previously utilized an internal reference: a constitutive *NOS* promoter driving the expression of RLUC. We showed that including RLUC on the same plasmid as the FLUC reporter, rather than on separate co‐expressed plasmids, led to a stronger and more consistent correlation between constitutively expressed RLUC and FLUC (Mortensen, Bernal‐Franco, et al., 2019). Here, we quantified the variability of raw FLUC, raw RLUC, and normalized FLUC/RLUC between replicate conditions and experiments, demonstrating the critical importance of utilizing a reference reporter for quantitative assays following *Agrobacterium*‐mediated transient transformations.

**FIGURE 4:**
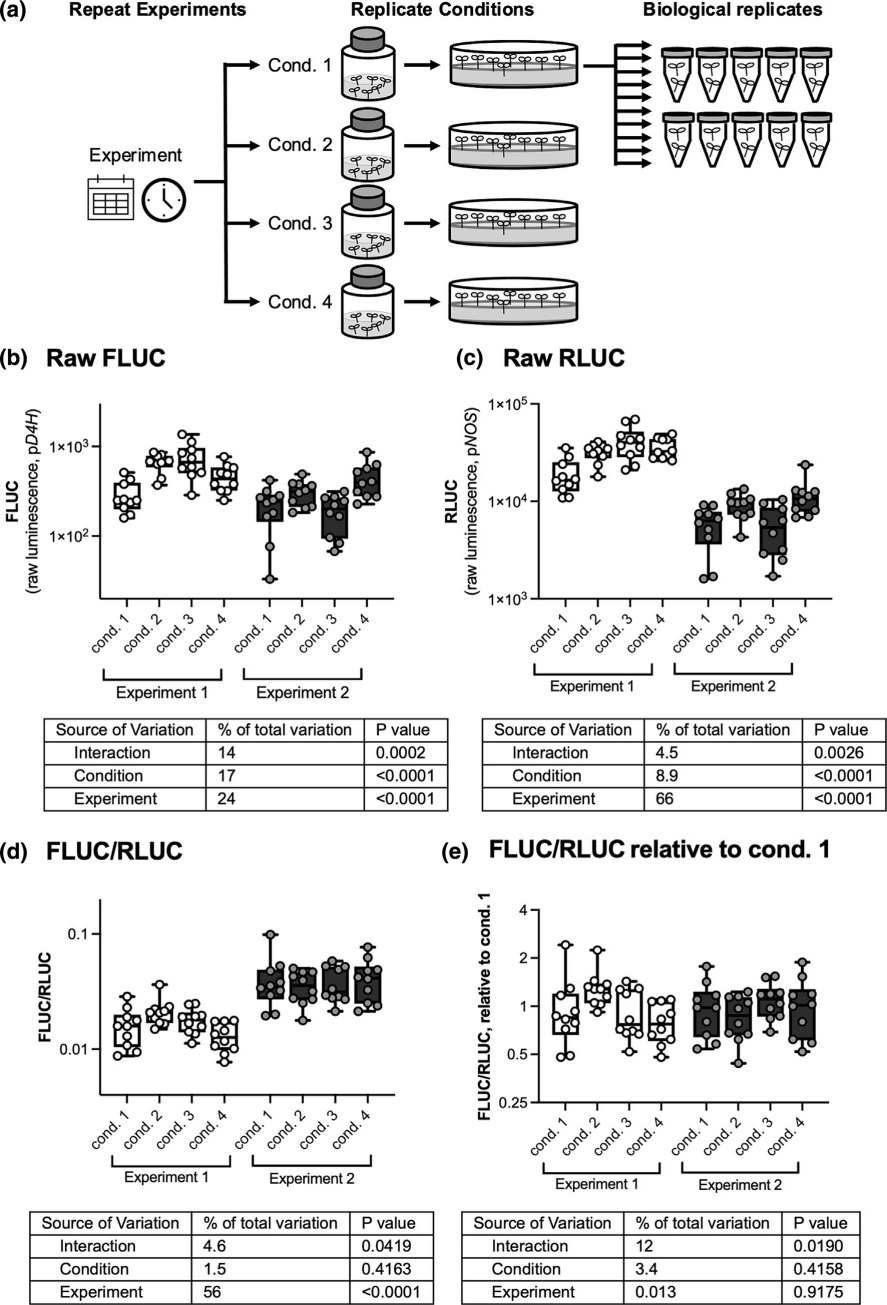
Normalization of EASI experiments to the RLUC reference reporter and to the control condition effectively reduced variability between replicate conditions within and between experiments. (a) In two independent experiments, four replicate conditions (cond.) were infiltrated with the same *agrobacterium* strains: (1) A reporter strain containing the *D4H* promoter (p*D4H*) driving firefly luciferase (*FLUC*) and the p*NOS* promoter driving *Renilla* luciferase (*RLUC*); (2) An effector strain expressing *GUS*. Each data point or biological replicate is a pool of two seedlings, *N* = 10 per experiment. (b,c) In both experiments, raw FLUC and RLUC luminescence values differed significantly between the four replicates. (d) Normalization of FLUC to RLUC removed variation between conditions within an experiment but did not remove variability between experiments. (e) Expressing normalized FLUC/RLUC values relative to Condition 1 of each experiment and taking the natural logarithm effectively removed the variability between experiments. % of total variation and *p* values are the result of a full‐factorial two‐way ANOVA on log‐transformed luminescence comparing the effects of replicate conditions and experimental repeats. Box plots represent the 25th and 75th percentile with a line marking the median. Whiskers extend to the minimum and maximum.

We measured FLUC and RLUC luminescence for four replicate conditions repeated in two experiments on different days (Figure 4a). For each replicate condition, seedlings were infiltrated with the same *Agrobacterium* strains mixed in a 1:1 ratio: a reporter strain containing both the p*D4H* promoter driving *FLUC* and the p*NOS* promoter driving *RLUC* and an effector strain expressing *GUS* (representing the negative effector control). We then applied a full‐factorial two‐way ANOVA to quantify the variation in luminescence between replicate conditions, repeat experiments, and the interaction between the two (interaction = condition * experiment). The interaction term indicates whether significant differences (*p* < .05) occurred between conditions in one but not in both experiments. We would expect the ANOVA to yield non‐significant *p* values between identical replicate conditions and identical repeat experiments; significant contributions of conditions and experiments to the overall variation observed (*p* < .05) suggest false positives.

Despite identical infiltration conditions, raw FLUC and RLUC luminescence values varied significantly between replicate conditions and between repeat experiments (Figure 4b,c, *p* < .0001 for source of variability = “condition” and “experiment”). Variability in raw FLUC and raw RLUC luminescence between replicate conditions contributed 9% to 17% of the total variation while variability between repeat experiments contributed 24% to 66% of the total variation. Effect sizes of up to 2.6‐fold were seen between replicate conditions in the same experiment.

Normalizing for transformation efficiency, FLUC divided by RLUC luminescence (FLUC/RLUC) successfully removed the significant variability between replicate conditions (Figure 4d, *p* = .42 for source of variability = “condition”). However, there was still a significant 1.2‐fold difference between two replicate conditions in Experiment 1 (Figure 4d, *p* < .05 for source of variability = “interaction”) and a significant two‐fold difference between the two experiments (Figure 4d, *p* < .0001 for source of variability = “experiment”).

To normalize results from multiple experiments, we divided the FLUC/RLUC values by the average FLUC/RLUC value for Condition 1 (arbitrarily chosen for this experiment but normally a negative control condition). This normalization removed the variation between the repeat experiments and allowed direct comparison of effect sizes between the two experiments (Figure 4e, *p* = .92 for source of variability = “experiment”). The interaction term remained significant (Figure 4e, *p* < .05 for source of variability = “interaction”), indicating that there was a significant difference between replicate conditions (i.e., a false positive) in one experiment but not in both. When both experiments were considered, there was no difference between the replicate conditions (Figure 4e, *p* = .42 for source of variability = “condition”). This highlighted the importance of repeating experiments to confirm effects and reduce false positives, especially for small effect sizes of less than 1.5‐fold.

After normalizing to RLUC and the negative control condition, variability associated with “condition,” “experiment,” and “interaction” accounted for 15% of total variation (Figure 4e). The remaining variation is from biological replicates within a replicate condition. This variability was consistent in both raw and normalized data. The coefficient of variation (CV) within a replicate condition was around 35% for raw FLUC, raw RLUC, and FLUC/RLUC values.

Finally, we determined that all these measurements (raw FLUC, raw RLUC, FLUC/RLUC, and FLUC/RLUC relative to Condition 1) benefited from a log‐transformation to increase the normality and homoscedasticity of the residuals following the two‐way ANOVA analysis (Figures S4 and S5). We recommend that luminescence measurements acquired from transient *Agrobacterium*‐mediated transformations be normalized and log‐transformed prior to statistical analysis to ensure that assumptions of normality and equal variances are met.

Overall, these results showed that the high variability associated with transient *Agrobacterium*‐mediated transformations can lead to false positives if appropriate normalization methods are not employed. However, normalization of a reporter (i.e., FLUC) to a reference reporter (i.e., RLUC) and experimental repetition can lead to robust quantitation of effector and promoter–reporter assays following *Agrobacterium*‐mediated transient transformation of plants.

## 4 DISCUSSION

In this paper, we experimentally determined critical parameters for improving the transformation efficiency of *Agrobacterium*‐mediated infiltration of *C. roseus* seedlings. Tested parameters included seedling developmental stage, *Agrobacterium* growth stage and cultivation, and dark incubations before and after *Agrobacterium* infiltration (Table 1). Our improved methodology can be used to study low‐expressing promoters in *C. roseus* and inform the optimization of *Agrobacterium*‐mediated transformation protocols in other plant species.

**TABLE 1:** Summary of parameters tested in this study and their effects on *agrobacterium*‐mediated transformation efficiency.

Parameter	Sensitivity	How to improve transformation efficiency
Seedling developmental stage	High	Young seedlings are much more susceptible to transformation than older seedlings. In *C. roseus*, seedlings should be germinated until just the radicle and apical hook have developed and then transferred to light until cotyledons emerge and turn green.
*Agrobacterium* growth stage and cultivation conditions	Low	*Agrobacteria* in exponential phase are slightly more virulent than those in stationary phase. *Agrobacterium* cultures can be initiated directly from glycerol stocks rather than from a single colony or a streak of colonies without reduction in transformation efficiency.
Pre‐infiltration and post‐infiltration dark incubations	High	Incubating seedlings overnight in the dark before and after *Agrobacterium* infiltration is critical for high transformation efficiency.
Natural variability in transformation and transgene expression	High	Natural variability in transformation and transgene expression can lead to significant differences between replicate conditions and replicate experiments. However, normalization by RLUC, normalization by negative control, and performing a second experiment reduces variability and minimizes false positives.

### 4.1 Seedling developmental stage

Literature repeatedly reports that young tissue is more efficiently transformed than mature tissue (Lu et al., 2017; Mortensen, Bernal‐Franco, et al., 2019; Peña et al., 2004). The molecular mechanisms responsible for this age‐related decrease in transformation efficiency are not understood; proposed mechanisms include the following: the development of cuticle wax, which physically blocks *Agrobacterium* from entering leaf tissue and inhibits infiltration (Shaheenuzzamn et al., 2019), the decrease in actively dividing cells when more efficient T‐DNA integration occurs (Peña et al., 2004), and the increased resistance to *Agrobacterium* infection with age (Hu & Yang, 2019). Consistent with literature, our results show that developmental state is one of the most critical parameters for high transformation success; just one additional day of germination led to a seven‐ to eight‐fold decrease in transformation efficiency (Figure 1). We thus recommend using seedlings (or other tissue types) as young as possible. For example, *C. roseus* seedlings should be transferred to the light when they are less than 1 cm in length, when only the radicle and apical hook have developed and before cotyledons emerge. The exact time required for seedlings to reach this stage will vary based on environmental conditions like temperature, humidity, seed batch, and planting method.

### 4.2 *Agrobacterium* growth stage and cultivation conditions

Many parameters relating to *Agrobacterium* growth have been optimized previously, such as a final OD_600_ = .4 for infiltration (Mortensen, Bernal‐Franco, et al., 2019), the GV3101 (pMP90) strain of *Agrobacterium* (Chetty et al., 2013), induction with acetosyringone and expression of a constitutively active mutated VirG (Mortensen, Bernal‐Franco, et al., 2019), and pre‐culture and infection media (Wu et al., 2014). In this paper, we determined whether there was flexibility in *Agrobacterium* growth timelines.

Previous *Agrobacterium*‐mediated transformation protocols have recommended using *Agrobacterium* in the exponential phase with an OD_600_ no greater than 1.5–2.0 (Colinas & Goossens, 2022; Fister et al., 2016; Sparkes et al., 2006). We showed that *Agrobacterium* in the exponential phase (OD_600_ = 1.0) increased the transformation efficiency of *C. roseus* seedlings by approximately two‐fold compared with *Agrobacterium* in the stationary phase (OD_600_ = 3.0). This is similar to a previous study that reported a two‐ to five‐fold increase in transformation efficiency of *Phaseolus acutifolius* calli with *Agrobacterium* grown into early‐ compared with late‐exponential growth (De Clercq et al., 2002). Researchers can decide whether this two‐fold increase in transformation is worth the additional labor of catching *Agrobacteria* in an exponential growth phase.

To further increase flexibility and reduce labor, we showed that liquid cultures of *Agrobacterium* could be inoculated directly from glycerol stocks rather than from either a single colony or a streak of colonies from a plate without reducing transformation efficiency. Overall, we showed that there is flexibility associated with *Agrobacterium* growth parameters, which should facilitate development of easy‐to‐use transformation protocols.

### 4.3 Pre‐ and post‐infiltration dark incubation

We showed that seedling incubation in the dark, both pre‐ and post‐infiltration, were critical for successful transformation, increasing transformation rates by five‐ to 13‐fold. Most *Agrobacterium*‐infiltration methods do not include a pre‐infiltration dark incubation (Acanda et al., 2021; Cao et al., 2017; Lu et al., 2017; Salazar‐González et al., 2023; Sparkes et al., 2006; Taak et al., 2020; Zhang et al., 2020). Our previous *Agro*infiltration method included a pre‐infiltration dark incubation based on the Lee and Yang protocol (Lee & Yang, 2006). To the best of our knowledge, we are the first to experimentally show that an overnight pre‐infiltration dark incubation of seedlings significantly enhanced transformation efficiency by seven‐ to 13‐fold.

In contrast to pre‐infiltration dark incubation, post‐infiltration dark incubations followed by light treatment are common but not standardized in *Agro*infiltration protocols (Lee & Yang, 2006; Salazar‐González et al., 2023; Taak et al., 2020; Zhang et al., 2020); many protocols contain different post‐infiltration light treatments (Acanda et al., 2021; Cao et al., 2017; Lu et al., 2017). The dark encourages *Agrobacterium* growth (Oberpichler et al., 2008) while light promotes transformation (Cazzonelli & Velten, 2006; De Clercq et al., 2002; Zambre et al., 2003). Recently, Zhang et al. (2020) experimentally showed that a post‐infiltration dark incubation followed by light significantly increased transient transformation efficiency in *Arabidopsis* leaves compared with incubation in only dark or only light. Our results confirmed that an overnight dark incubation followed by light treatment significantly enhanced transformation efficiency by five‐ to seven‐fold, suggesting that this should be adopted and standardized in *Agro*infiltration protocols.

### 4.4 Quantitative validation

Lastly, we quantified the variation in our EASI method within a given condition, between replicate conditions, and between replicate experiments. Within a given condition, we observed a CV of about 35% for raw FLUC, raw RLUC, or normalized FLUC/RLUC values. This is similar to a previous study quantifying variation in reporter levels in transiently transformed strawberry fruit; they reported CVs of 58% for raw RLUC, 78% for raw FLUC, and 39% for normalized RLUC/FLUC values (Zeng et al., 2021). Between replicate conditions, we observed significant variability in raw FLUC and RLUC values, contributing up to 17% of total variation and significant differences of up to 2.6‐fold between replicate conditions. Similarly, Bashandy et al. (2015) observed in *N. benthamiana* leaves that the greatest source of variation of raw FLUC came from replicate infiltrations within a single leaf. Although a second reporter gene is often employed in *Agrobacterium*‐mediated transformation assays to normalize for transformation efficiency, few studies have confirmed that this strategy reduces variation. Here, we confirmed that normalization of FLUC to RLUC successfully removed variation between replicate conditions, reducing its contribution to total variation to 1.5% and removing significant differences between replicate conditions. We have thus confirmed that *Agrobacterium*‐mediated transient transformations can be used for accurate quantitative analyses, although experimental repetition is still important to validate very small effect sizes (less than 1.5‐fold).

## 5 CONCLUSION

*Agrobacterium*‐mediated transient transformations have become a common tool for plant biologists but still suffer from variable and low transformation efficiencies. Towards more standardized and robust transformations, we experimentally characterized parameters influencing transformation efficiency of *C. roseus* seedlings. We determined that critical parameters influencing transformation efficiency include the plant developmental state and pre‐ and post‐infiltration dark incubations while the *Agrobacterium* growth stage and cultivation methods were less critical. Importantly, we showed for the first time that a pre‐infiltration dark incubation of the seedlings increased transformation efficiency by seven‐ to 13‐fold. Additionally, we showed that normalization of the FLUC reporter to the internal RLUC reference effectively reduced variation and false positives between replicate infiltrations, strengthening the credibility of utilizing transient transformations for quantitative hypothesis testing. This study and the resulting standardization of *Agrobacterium‐*mediated transient transformation methods will facilitate the exploration of gene function in both model and non‐model plant species, like *C. roseus*.

## AUTHOR CONTRIBUTIONS

Lauren F. Cole‐Osborn and Carolyn W. T. Lee‐Parsons designed the experiments and wrote the manuscript. Lauren F. Cole‐Osborn and Emma Meehan performed the experiments and all authors analyzed the data. All authors edited and approved the manuscript.

## CONFLICT OF INTEREST STATEMENT

The authors have no relevant financial or non‐financial interests to disclose.

## Supporting information


**Figure S1.** Seedlings pushed into solid agar media to varying levels germinate at different rates.

**Figure S2.** Relative promoter activity of p*ZCT1*, p*STR*, and p*D4H*.

**Figure S3.** T‐DNA regions of plasmids used in this paper.

**Figure S4.** Quantile‐quantile (Q‐Q) plots.

**Figure S5.** Homoscedasticity plots.

**Table S1.** Cloning primers.

**Data S1.** Peer Review.

## Data Availability

The *pD4H* reporter and *GUS* effector plasmids are available from Addgene (IDs: 203901 and123197, respectively). Datasets generated during the current study are available from thecorresponding author on reasonable request.
