# Supplementary figures and images for: Critical parameters for robust Agrobacterium‐mediated transient transformation and quantitative promoter assays in Catharanthus roseus seedlings
Source: Plant Direct. 2024 Jun 5;8(6):e596. doi: 10.1002/pld3.596 (PMC11154794; doi:10.1002/pld3.596)

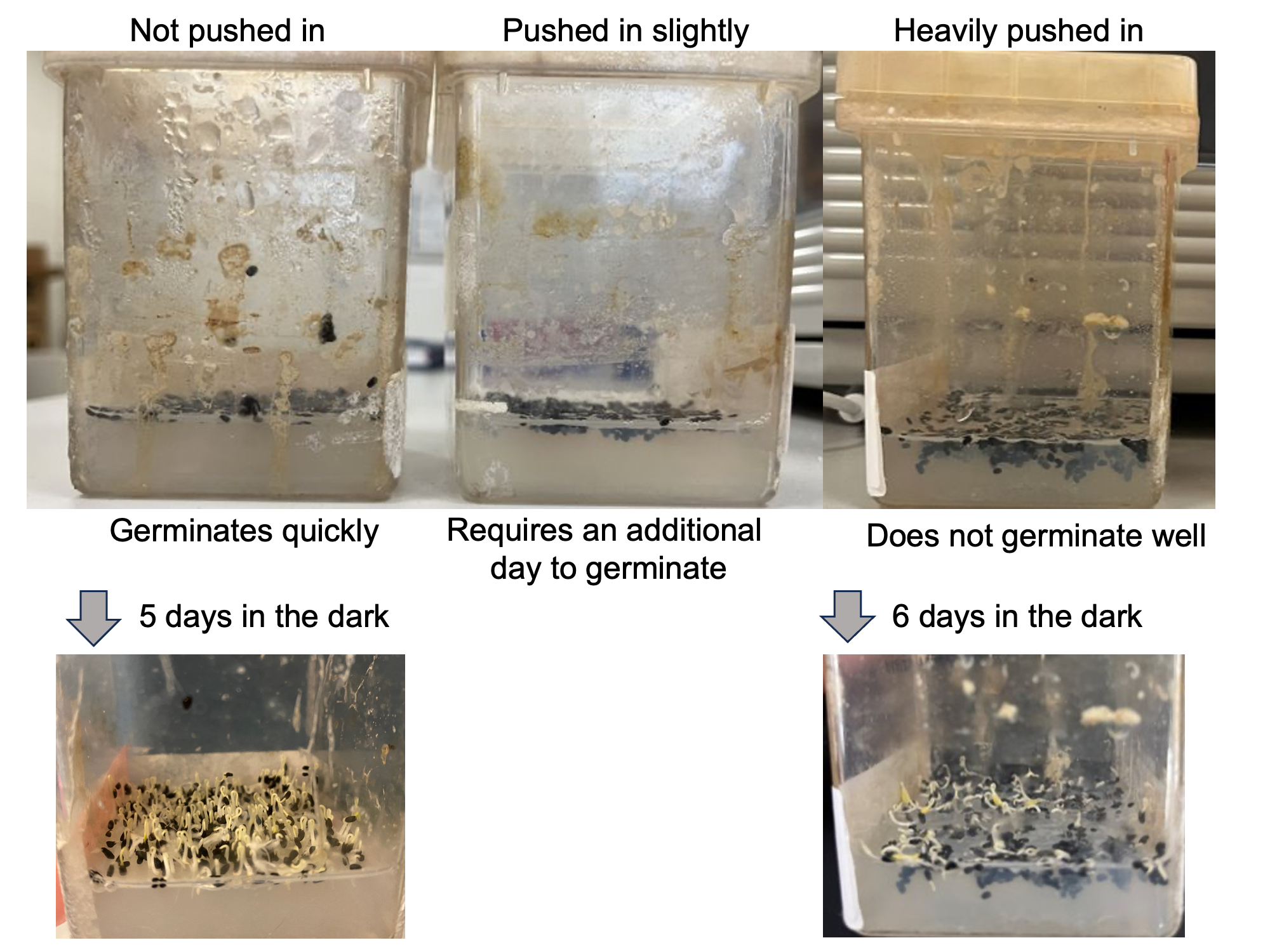

Supplement: Supplementary file 1 — Figure S1. Seedlings pushed into solid agar media to varying levels germinate at different rates. [file PLD3-8-e596-s007.tiff]

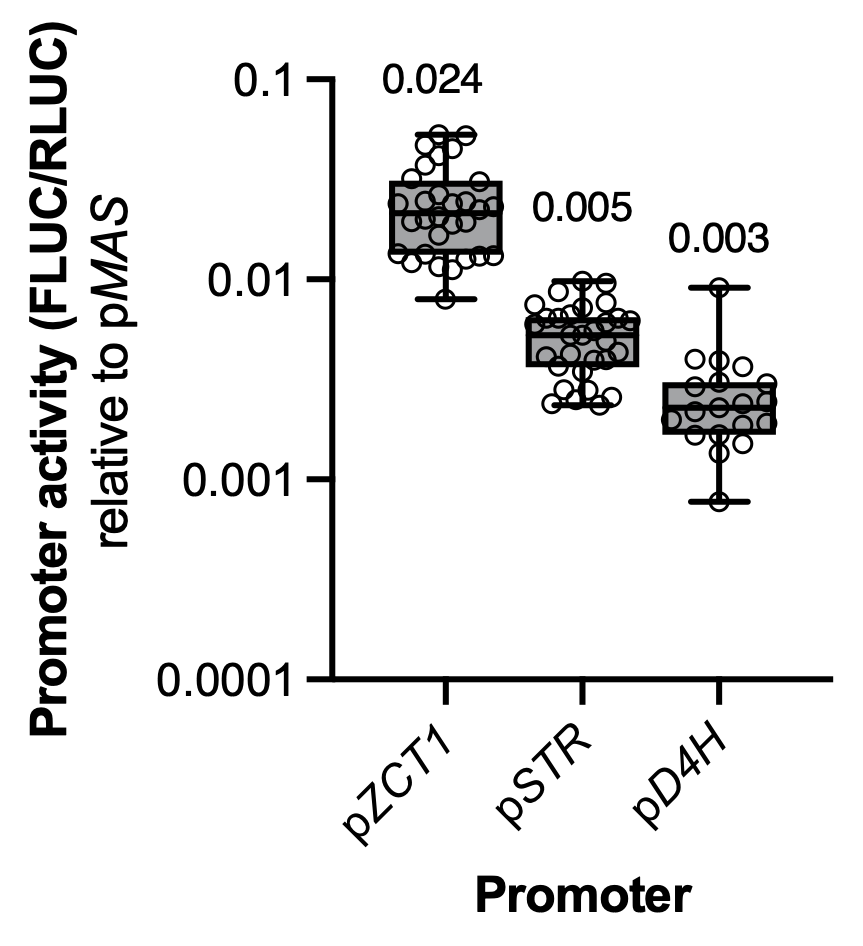

Supplement: Supplementary file 2 — Figure S2. Relative promoter activity of pZCT1, pSTR, and pD4H. [file PLD3-8-e596-s003.tiff]

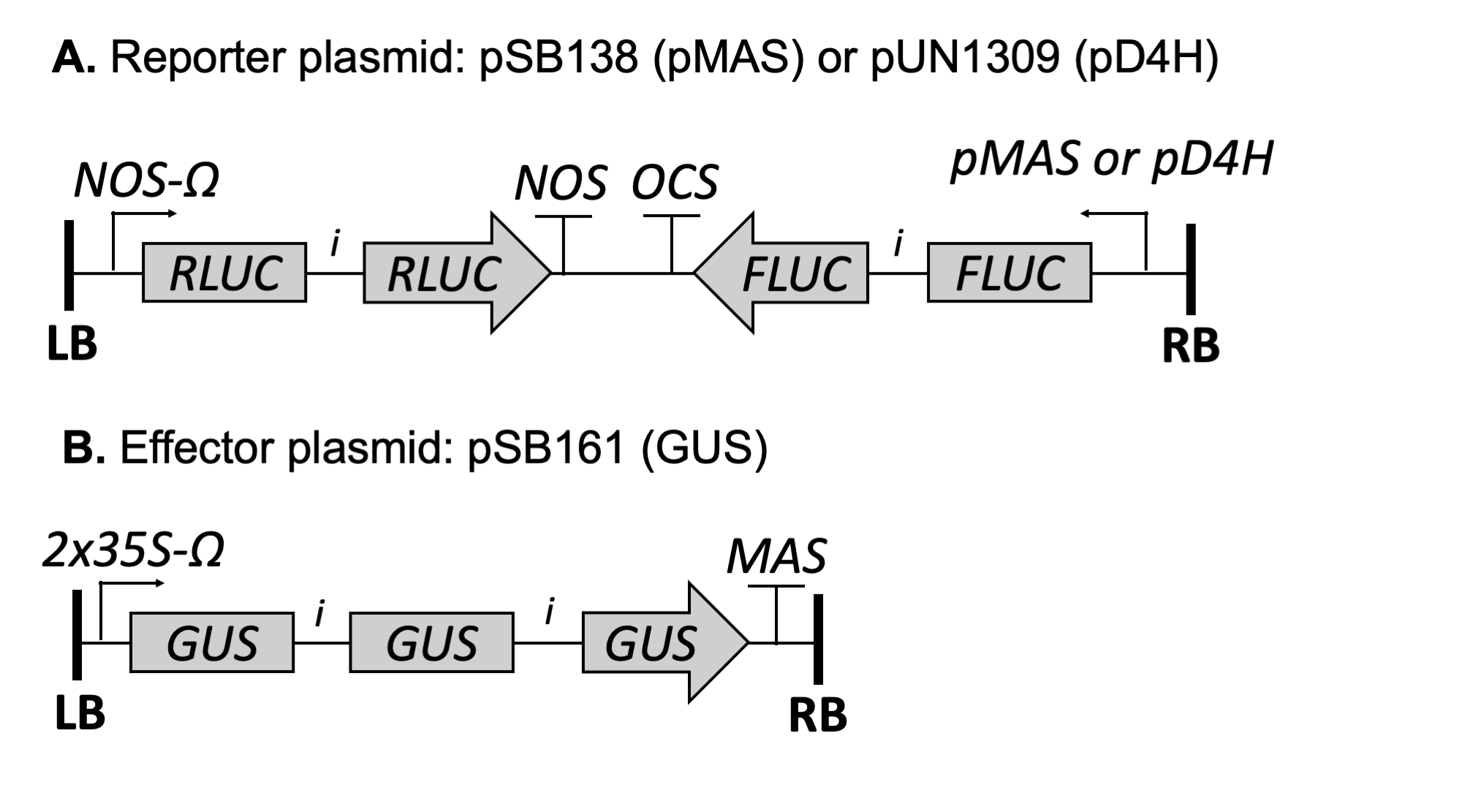

Supplement: Supplementary file 3 — Figure S3. T‐DNA regions of plasmids used in this paper. [file PLD3-8-e596-s001.tiff]

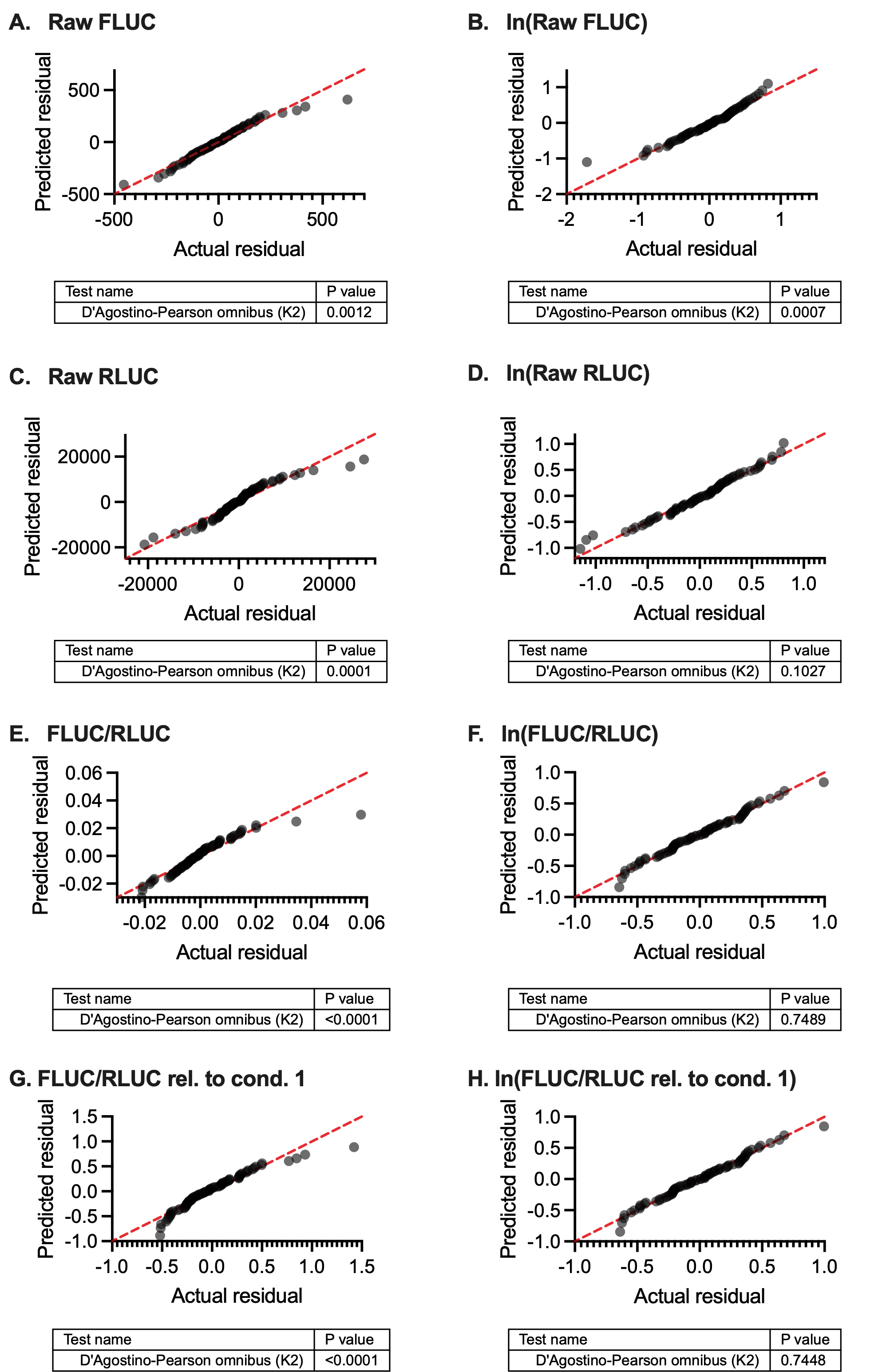

Supplement: Supplementary file 4 — Figure S4. Quantile‐quantile (Q‐Q) plots. [file PLD3-8-e596-s006.tiff]

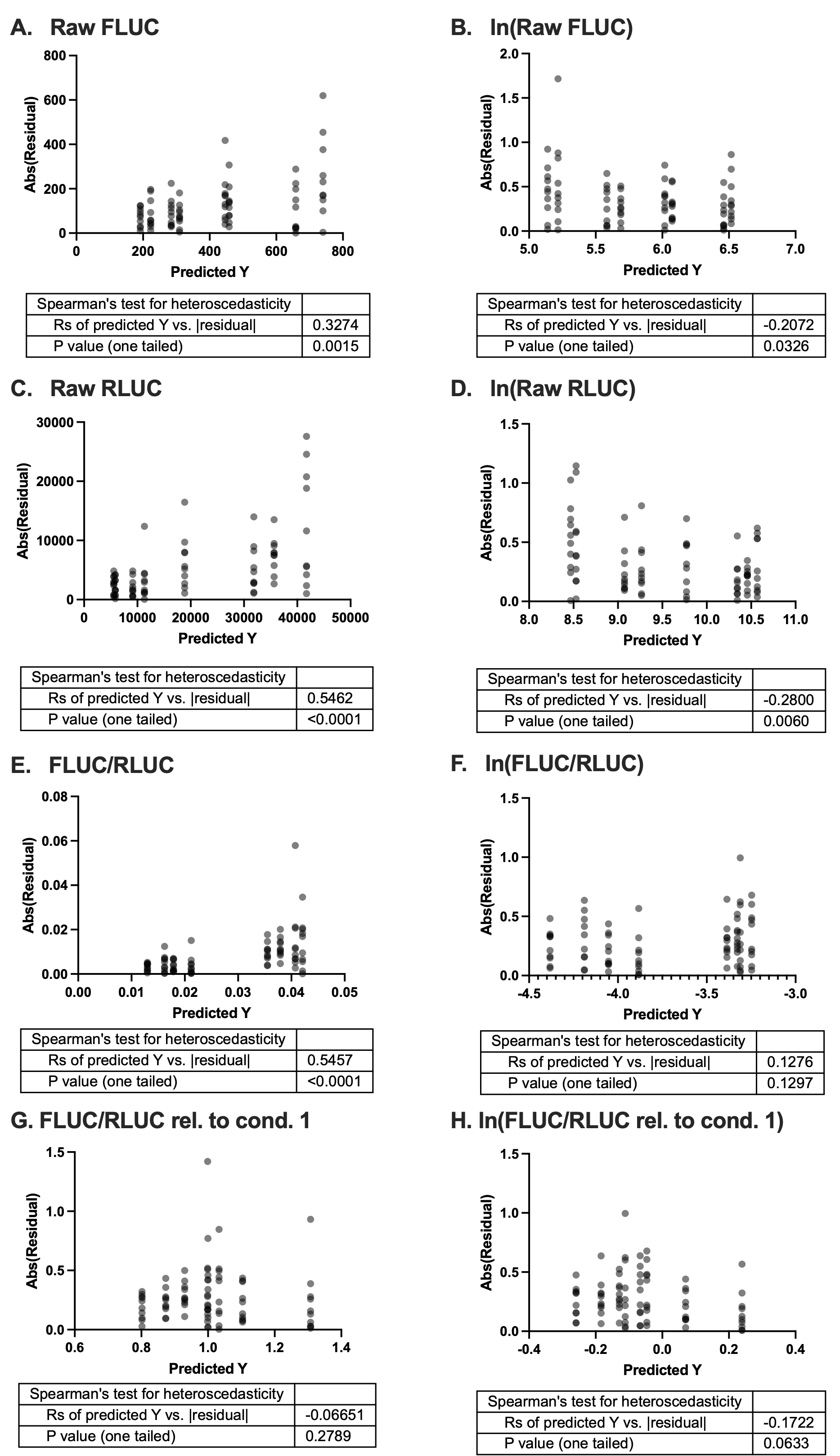

Supplement: Supplementary file 5 — Figure S5. Homoscedasticity plots. [file PLD3-8-e596-s004.tiff]
