# Supplementary material for: Critical parameters for robust Agrobacterium‐mediated transient transformation and quantitative promoter assays in Catharanthus roseus seedlings
Source: Plant Direct. 2024 Jun 5;8(6):e596. doi: 10.1002/pld3.596 (PMC11154794; doi:10.1002/pld3.596)
Supplement: Supplementary file 6 — Table S1. Cloning primers. [file PLD3-8-e596-s002.docx]

**Supplemental Material**

Critical parameters for robust *Agrobacterium*-mediated transient transformation and quantitative promoter assays in *Catharanthus roseus* seedlings

Lauren F. Cole-Osborn, Emma Meehan, Carolyn W.T. Lee-Parsons

Table S1. Cloning primers.

Uppercase indicates sequences complementary to its target while lowercase indicates 5’ overhangs to facilitate cloning.

| **Primer Name** | **Primer Sequence** | **Purpose** |
| --- | --- | --- |
| D4H_P_F1_2 | aagaagacaaggagTATTTCTTCTCACTAATAATAATTAATTTCATATTGTTC | Amplification of *D4H* Promoter |
| D4H_P_R1_2 | aagaagacaacatTTTTCTTTCTTGCTCAGAATTTGG |  |


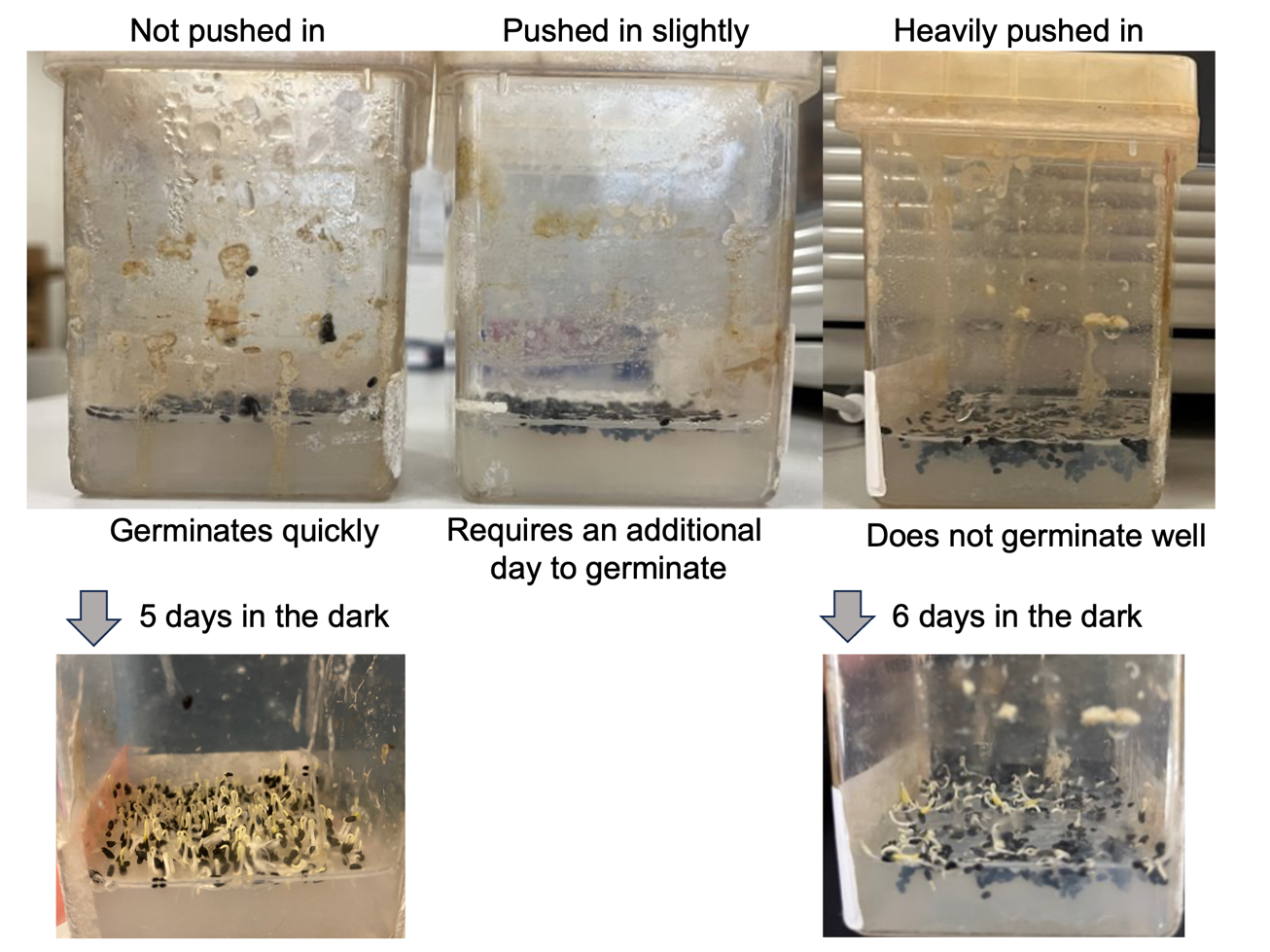


**Figure S1.** Seedlings pushed into solid agar media to varying levels germinate at different rates.


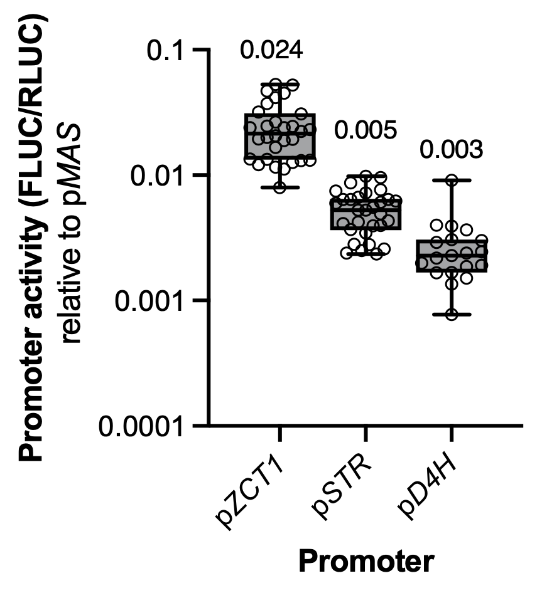


**Figure S2.** **Relative promoter activity of p*ZCT1*, p*STR*, and p*D4H***, compared to the highly expressed p*MAS* promoter. p*ZCT1*, p*STR*, p*D4H*, or p*MAS* drove expression of firefly luciferase (FLUC) while a constitutive *AtuNOS* promoter drove expression of *Renilla* luciferase (RLUC). Promoter activity was calculated as FLUC normalized to RLUC (FLUC/RLUC), relative to FLUC/RLUC of the strong *MAS* promoter. Investigation of the p*ZCT1* and p*STR* activities consisted of 3 experimental repeats (10 biological replicates per experimental repeat, N = 30) while investigation of the p*D4H* activity consisted of two experimental repeats (N = 20). Each data point or biological replicate is a pool of 2 seedlings. Box plots represent the 25^th^ and 75^th^ percentile with a line marking the median. Whiskers extend to the minimum and maximum. The number above each boxplot is the mean.


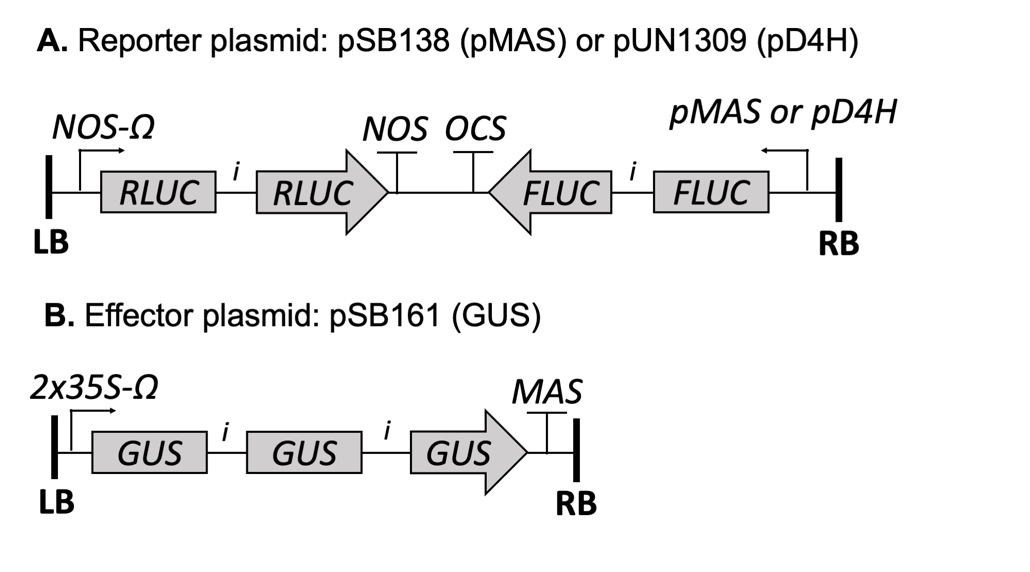


**Figure S3. T-DNA regions of plasmids used in this paper.** Seedlings were infiltrated with two *Agrobacterium tumefaciens* strains in a 1:1 ratio. One strain contained a reporter plasmid and the other strain contained an effector plasmid. (A) The reporter plasmid consists of the *AtuNOS* promoter, TMV Ω 5’UTR, and *AtuNOS* terminator driving the expression of intron-containing *Renilla* luciferase (*RLUC*) and either the *MAS* or *D4H* promoter (p*MAS* or p*D4H*) and OCS terminator driving the expression of intron-containing firefly luciferase (*FLUC*). (B) The effector plasmid consists of the *CaMV* 2x35S promoter, TMV Ω 5’UTR, and *AtuMAS* terminator driving the expression of intron-containing *beta-glucuronidase* (*GUS*). LB and RB indicate the left and right borders of the *Agrobacterium* T-DNA; *i* indicates an intron
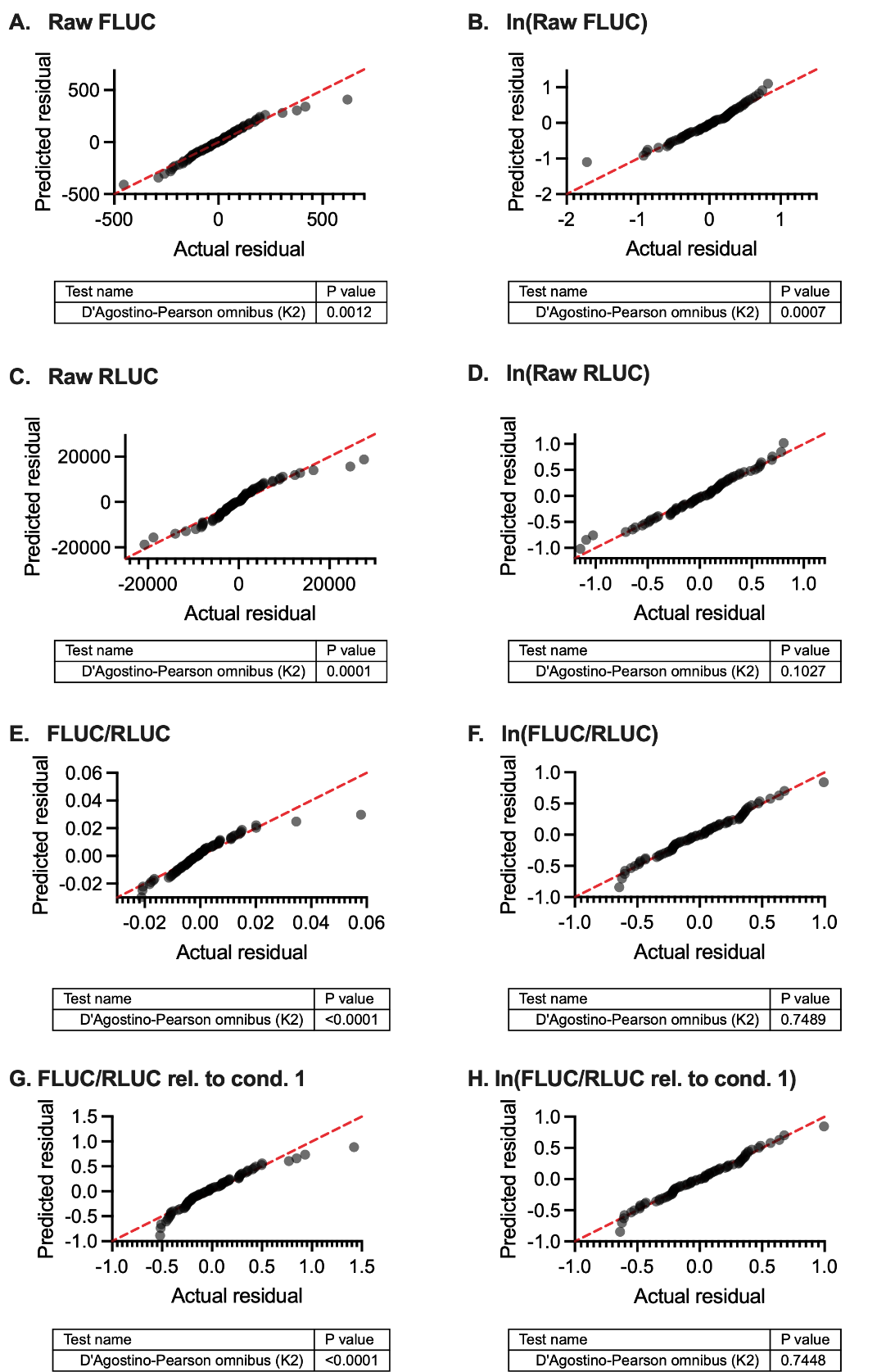


**Figure S4. Quantile-quantile (Q-Q) plots** for normal and lognormal distribution of residuals following a two-way ANOVA of raw or log-transformed data from the experiments reported in Figure 4. “Predicted residual” (y-axis) represents the residual predicted given a normal distribution while “actual residual” (x-axis) represents the actual spread of the data. A linear response with a slope of 1 indicates that the data follows a normal distribution while the D’Agostino-Pearson omnibus (K2) tests the hypothesis that the spread of the residuals does not follow a normal distribution. The raw linear data (A, C, E, G) have distributions that significantly differ from the normal distribution as indicated by p < 0.05 in the D’Agostino-Pearson omnibus (K2) test and a curve in the Q-Q plots. In contrast, log-transformed data (B, D, F, H) is mostly normal as indicated by p > 0.05 in the D’Agostino-Pearson omnibus (K2) test and a linear Q-Q plot.


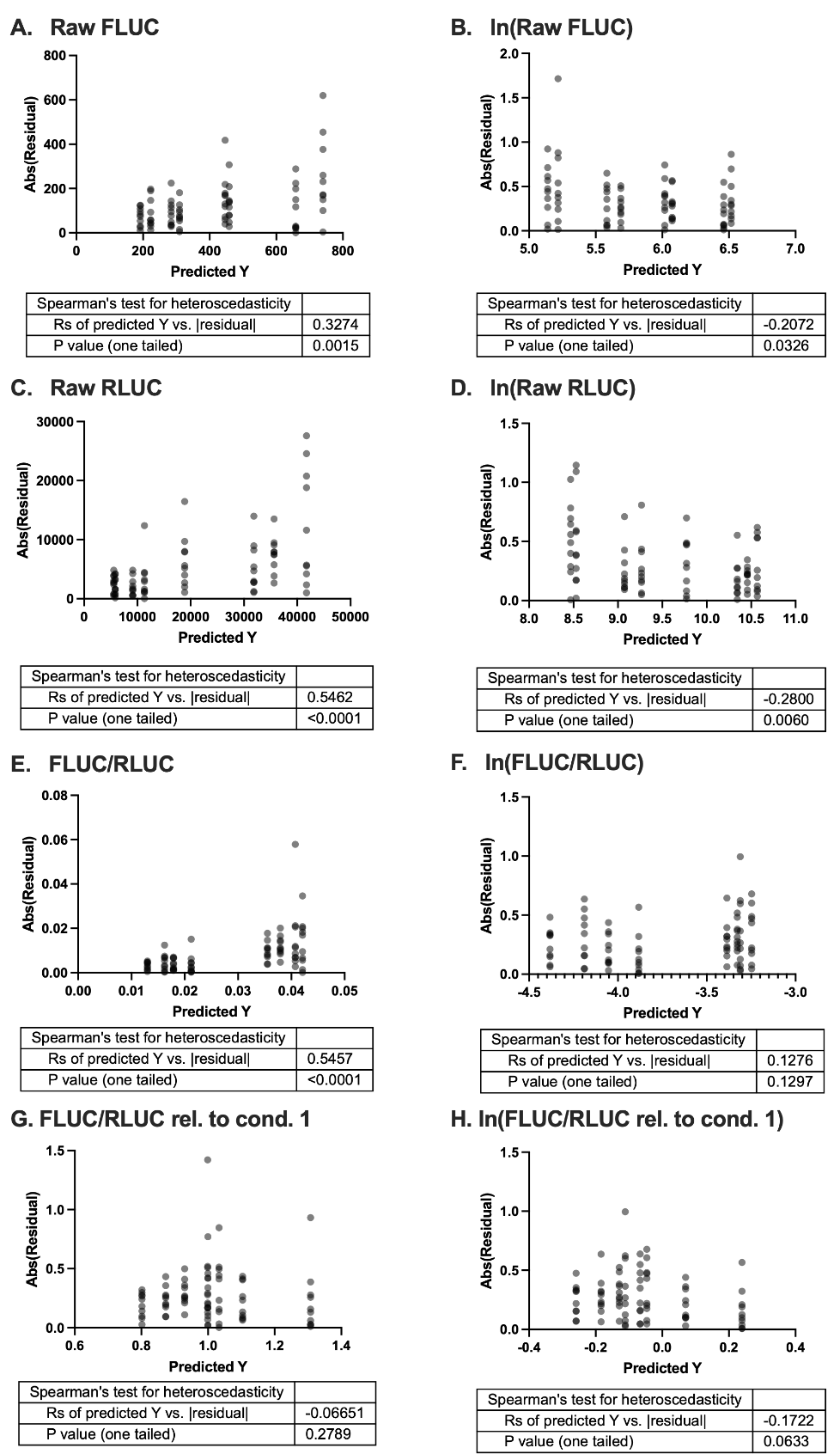


**Figure S5. Homoscedasticity plots** of residuals following a two-way ANOVA of raw or log-transformed data from the experiments reported in Figure 4. The “predicted Y” (x-axis) from the two-way ANOVA is equivalent to the mean for each condition. The distance of each data point (i.e. biological replicate) from the predicted mean is the residual. If variation is equivalent between conditions, there will be no correlation between the “predicted Y” and the “abs(residual)” (y-axis), indicated by an R value close to 0. Spearman’s test for heteroscedascity tests the hypothesis that variance is unequal between groups. A positive correlation between abs(residual) and predicted y indicates heteroscedasticity or unequal variances between groups. The raw linear data (A, C, E, G) tended to have greater heteroscedascity than log-transformed data (B, D, F, H).
