# Supplementary material for: Critical parameters for robust Agrobacterium‐mediated transient transformation and quantitative promoter assays in Catharanthus roseus seedlings
Source: Plant Direct. 2024 Jun 5;8(6):e596. doi: 10.1002/pld3.596 (PMC11154794; doi:10.1002/pld3.596)
Supplement: Supplementary file 7 — Data S1. Peer Review. [file PLD3-8-e596-s005.pdf]

Plant Direct
Open Access

Critical parameters for robust *Agrobacterium*-mediated transient transformation and quantitative promoter assays in *Catharanthus roseus* seedlings

Lauren F. Cole-Osborn[1,2], Emma Meehan[1,3], Carolyn W.T. Lee-Parsons[1,4,5,6,7]

Decision Letter Round 1:
April 2, 2024

Prof. Carolyn W. Lee-Parsons
Northeastern University
Chemistry & Chemical Biology and Chemical Engineering
313 Snell Engineering Center
360 Huntington Ave
Boston, Massachusetts 02115

RE: Critical parameters for robust Agrobacterium-mediated transient transformation and quantitative promoter assays in Catharanthus roseus seedlings

Dear Dr. Lee-Parsons:

Thank you for submitting to Plant Direct. All required reviews have been returned and we have now finished our evaluation of your manuscript. In light of the reviewers' and editor's comments, further revisions are needed before the paper can be accepted for publication in Plant Direct.

Please view the editors' and reviewers' comments below and use their suggestions as a guide while you work on your revision.

When uploading the revised version of this article, please be sure to include the following:

-A word document that contains your response to the reviewers. You should respond to each reviewer comment and note the changes made to the manuscript. If you do not agree with a reviewer's comment and choose not to make a suggested revision, please explain why. Please try to provide as complete an answer as possible to each reviewer's criticisms.
-A tracked changes document with each change highlighted
- A clean version of the latest version of the manuscript

To upload your revision, please click the link below.
https://plantdirect.msubmit.net/cgi-

bin/main.plex?el=A2Lr6gh1A7mZV2I6A9ftdogxGuvVeUCLcqjCKDk44KgZ

In order to provide as timely a service as possible, we ask that your revision is resubmitted within three months after receipt of this request. If an extension is needed, please send a request, along with a brief explanation, to the editorial office at plantdirect@wiley.com .

Please note that, in addition to publishing reviewer comments, the author's responses to review comments will also be published alongside the final version of the paper. If you would not like the author's responses to be published, please contact the editorial office at plantdirect@wiley.com .

Thank you very much for giving us an opportunity to review your work. I look forward to receiving the next version.

Sincerely,

Eva Farre

Ana Fortes

Editor, Plant Direct

-- Editor comments:

-- Reviewer comments:
Reviewer #1:

Dear Editor, In the present work, the authors have performed experiments to further refine the transient transformation and expression in Catharanthus roseus seedlings. As such they have demonstrated the young seedlings are more amenable to be transformed than older seedlings and dark treatment pre and post Agrobacterium inoculations is important for higher transformation efficiencies. One of the interesting experiments is the quantitative validation of the transient transformations where the authors performed replicate experiments having four replicate conditions and recommended that the luminescence measurements should be normalized, and log transformed to remove the false positive and variability associated with variations in transformation efficiencies. Overall, the study is well designed, and experiments are well performed. However, there are few questions that need to be clarified: 1. In the transient transformation assay how many seedlings were transformed in each experiment? Does every time all the seedlings get transformed? Or is there any screening process before selection of two

seedlings as one replicate? 2. The authors in here test several parameters such as seedling growth parameters for 5 versus 6 days dark germination and 2 versus 3 days in light post germination (Figure 1). In both experiments it's not clear if the authors have subjected the seedlings to pre and post incubation dark treatments? If so, then how this experiment is different from experiment 3 (Figure 3) where the authors specifically test for dark treatments around incubation times. I don't see much difference in the raw transformation efficiencies of pD4H-FLUC and pNOS-RLUC in figure 1D (2d in light) and figure 3a or figure 3c. 3. In Figure 2, what were the conditions in which the seedlings were grown for the luciferase assay? And if the seedlings were subjected to any dark treatments pre and post Agrobacterium incubations. 4. Basically, in figures 1-3, the confusion is if the improvements in transformation efficiency in each experiment is comparable to each other or if it's just refinement of the various parameters around a basic skeleton of protocol. 5. In Figure 4, the authors mentioned that they used four replicate conditions in an experiment. But they do not mention what is the condition they have used for their experiment in terms of seedling growth and dark incubations. The term replicate conditions is a bit confusing. Does it mean four replicates of a single experimental condition (such as seedling growth and incubation times with agrobacterium).

Reviewer #2:

Cole-Osborn et al. have optimized parameters for Catharanthus roseus transient transformation by agrobacterium. The method has been set up and used by the same research group earlier and relies on vacuum, agroinfiltration of young seedlings. Here they optimize a set of parameters for the process, namely the seedling age for the assay, dark incubation periods before and after infiltration, and the agrobacterium culturing process. The latter proved to be a robust parameter, but both seedling stage and dark incubation affected significantly. They also evaluated infiltration normalization by using a second reporter (RUC - the primary reporter is LUC) and found that this reduced variation significantly.

The work is well performed and documented in a very readable for (although quite lengthy). As such, the results are probably Catharanthus specific, although they serve as a guide in choosing parameters for optimization also for other plants. An interesting detail that is not mentioned is how efficient Catharanthus transient assay is, compared to the much-used Nicotiana benthamiana assay.

In the introduction, the authors state that the T-DNA integrates (randomly) in the genome before transient expression. In my understanding this is not at all clear. The current view is that the T-strand that enters the cell is made double stranded before integration, allowing transient expression independently of integration. The claim the authors make needs a reference.

On page 6, line 2, it should be mentioned that the agro GV3101(pMP90) also harbors a binary T-DNA vector.

Decision Letter Round 2:

Plant Direct
Open Access

April 25, 2024

Prof. Carolyn W. Lee-Parsons
Northeastern University
Chemistry & Chemical Biology and Chemical Engineering
313 Snell Engineering Center
360 Huntington Ave
Boston, Massachusetts 02115

MSID: 2024-01450R1
MS TITLE: Critical parameters for robust Agrobacterium-mediated transient transformation and quantitative promoter assays in Catharanthus roseus seedlings

Dear Dr. Carolyn Lee-Parsons:

I am pleased to inform you that your manuscript "Critical parameters for robust Agrobacterium-mediated transient transformation and quantitative promoter assays in Catharanthus roseus seedlings" has been accepted for publication in Plant Direct.

Your article will appear online in the next available issue of Plant Direct. To ensure your article gets published as quickly as possible, please pay attention to the steps detailed below. We have found that most of the delays happen at this stage, especially at the payment stage, so please respond as quickly as possible when prompted.

License Agreement: Once your article has been accepted it will move to Production and undergo admin and file checking - you may receive an email with any queries we have at this stage. When all required items are received by the publisher and queries resolved, the corresponding author will receive an email from Wiley's Author Services system which will ask them to log on at https://authorservices.wiley.com/bauthor and will present them with the appropriate license for completion. Your article cannot be published until both the signed license agreement and payment of the article fee have been received.

Payment of the Open Access Article Publication Fee: All articles published in Plant Direct are fully open access: immediately and freely available to read, download and share. Plant Direct charges a publication fee to cover the publication costs. The corresponding author for this manuscript should have already received a quote from the payments team (cs-openaccess@wiley.com) with the estimated article publication fee; please email cs-openaccess@wiley.com if this has not been received. The corresponding author should log on to the Wiley Author Services site, where the publication fee can be paid by credit card or an invoice. Pro Forma can also be requested. Payment of the publication charge must be received before the article will be published online.

Proofs: You will have the opportunity to look over your paper once more when you receive the

author proofs for your article. The proofs will be with you in approximately two weeks. Please note that, in addition to publishing reviewer comments, the author's responses to review comments will also be published alongside the final version of the paper. If you would not like the author's responses to be published, please contact the editorial office at plantdirect@wiley.com .

Promotion of your article: You can help your research get the attention it deserves! Check out Wiley's free Promotion Guide for best-practice recommendations for promoting your work at www.wileyauthors.com/eeo/guide. And learn more about Wiley Editing Services which offers professional video, design, and writing services to create shareable video abstracts, infographics, conference posters, lay summaries, and research news stories for your research at www.wileyauthors.com/eeo/promotion.

Thank you again for your contribution to Plant Direct. If you have any questions, feel free to contact the editorial office at plantdirect@wiley.com .

Sincerely,

Eva Farre

Ana Fortes

Editor, Plant Direct

Author Response:

Reviewer #1: Dear Editor, In the present work, the authors have performed experiments to further refine the transient transformation and expression in Catharanthus roseus seedlings. As such they have demonstrated the young seedlings are more amenable to be transformed than older seedlings and dark treatment pre and post Agrobacterium inoculations is important for higher transformation efficiencies. One of the interesting experiments is the quantitative validation of the transient transformations where the authors performed replicate experiments having four replicate conditions and recommended that the luminescence measurements should be normalized, and log transformed to remove the false positive and variability associated with variations in transformation efficiencies. Overall, the study is well designed, and experiments are well performed. However, there are few questions that need to be clarified: 1. In the transient transformation assay how many seedlings were transformed in each experiment? Does every time all the seedlings get transformed? Or is there any screening process before selection of two seedlings as one replicate? • In each assay, we transform about 30-50 seedlings per group/condition, replant 30 seedlings, and then harvest 20 seedlings (i.e. ten biological replicates of two pooled seedlings). There is no screening process prior to selection, although any seedlings that have died during the process are generally avoided. When we do a qualitative GUS staining, we see that approximately 100% of seedlings have some amount of blue staining, indicating transformation, but the extent of the transformation can vary significantly. We have added this

additional information to the methods section on page 6. 2. The authors in here test several parameters such as seedling growth parameters for 5 versus 6 days dark germination and 2 versus 3 days in light post germination (Figure 1). In both experiments it's not clear if the authors have subjected the seedlings to pre and post incubation dark treatments? If so, then how this experiment is different from experiment 3 (Figure 3) where the authors specifically test for dark treatments around incubation times. I don't see much difference in the raw transformation efficiencies of pD4H-FLUC and pNOS-RLUC in figure 1D (2d in light) and figure 3a or figure 3c. 3. In Figure 2, what were the conditions in which the seedlings were grown for the luciferase assay? And if the seedlings were subjected to any dark treatments pre and post Agrobacterium incubations. 4. Basically, in figures 1-3, the confusion is if the improvements in transformation efficiency in each experiment is comparable to each other or if it's just refinement of the various parameters around a basic skeleton of protocol. • In response to points 2-4: The experiments are a refinement around a basic protocol rather than a progressive optimization. To clarify this, we added a description in the first paragraph of the results section, describing the parameters that were held constant while a single parameter was varied. • We see significant variability in transformation efficiency from experiment to experiment (as evidenced in Figure 4) so raw values should not be compared between experiments. Each of the parameters were tested in separate experiments (i.e. on different days). 5. In Figure 4, the authors mentioned that they used four replicate conditions in an experiment. But they do not mention what is the condition they have used for their experiment in terms of seedling growth and dark incubations. The term replicate conditions is a bit confusing. Does it mean four replicates of a single experimental condition (such as seedling growth and incubation times with agrobacterium). • We added additional information on pages 16-17 for clarification. Reviewer #2: Cole-Osborn et al. have optimized parameters for Catharanthus roseus transient transformation by agrobacterium. The method has been set up and used by the same research group earlier and relies on vacuum, agroinfiltration of young seedlings. Here they optimize a set of parameters for the process, namely the seedling age for the assay, dark incubation periods before and after infiltration, and the agrobacterium culturing process. The latter proved to be a robust parameter, but both seedling stage and dark incubation affected significantly. They also evaluated infiltration normalization by using a second reporter (RUC - the primary reporter is LUC) and found that this reduced variation significantly. The work is well performed and documented in a very readable for (although quite lengthy). As such, the results are probably Catharanthus specific, although they serve as a guide in choosing parameters for optimization also for other plants. An interesting detail that is not mentioned is how efficient Catharanthus transient assay is, compared to the much-used Nicotiana benthamiana assay. • In response to: how efficient is C. roseus assay compared to N. benthamiana assay?: We agree that this is an interesting question. However, we're not sure how to answer it because you can't directly compare relative light units (RLUs) between different instruments or studies. With that being said, a quick comparison suggests that our RLUs are on a similar order of magnitude as N. benthamiana assays. In our paper, FLUC and RLUC was around 1E4 – 1E5 RLUs for strong promoters (pMAS and pNOS). In (Moyle et al., 2017), RLUC driven by the NOS promoter (in N. benthamiana) was around 1E5 RLU. Moyle, R. L., Carvalhais, L. C., Pretorius, L.-S., Nowak, E., Subramaniam, G., DaltonMorgan, J., & Schenk, P. M. (2017). An Optimized Transient Dual Luciferase Assay for Quantifying MicroRNA Directed Repression of Targeted Sequences. Frontiers in Plant Science, 8, 1631.

Plant Direct
Open Access

https://doi.org/10.3389/fpls.2017.01631 • In the introduction, the authors state that the T-DNA integrates (randomly) in the genome before transient expression. In my understanding this is not at all clear. The current view is that the T-strand that enters the cell is made double stranded before integration, allowing transient expression independently of integration. The claim the authors make needs a reference. o Thank you, we have made this correction and added citations to the introduction. • On page 6, line 2, it should be mentioned that the agro GV3101(pMP90) also harbors a binary T-DNA vector. o Thank you for catching this, we have added this clarification.
